# Supplementary material for: A PPP-type pseudophosphatase is required for the maintenance of basal complex integrity in Plasmodium falciparum
Source: Nat Commun. 2023 Jul 3;14:3916. doi: 10.1038/s41467-023-39435-z (PMC10317984; doi:10.1038/s41467-023-39435-z)
Supplement: Supplementary file 1 — Supplementary Information [file 41467_2023_39435_MOESM1_ESM.pdf]

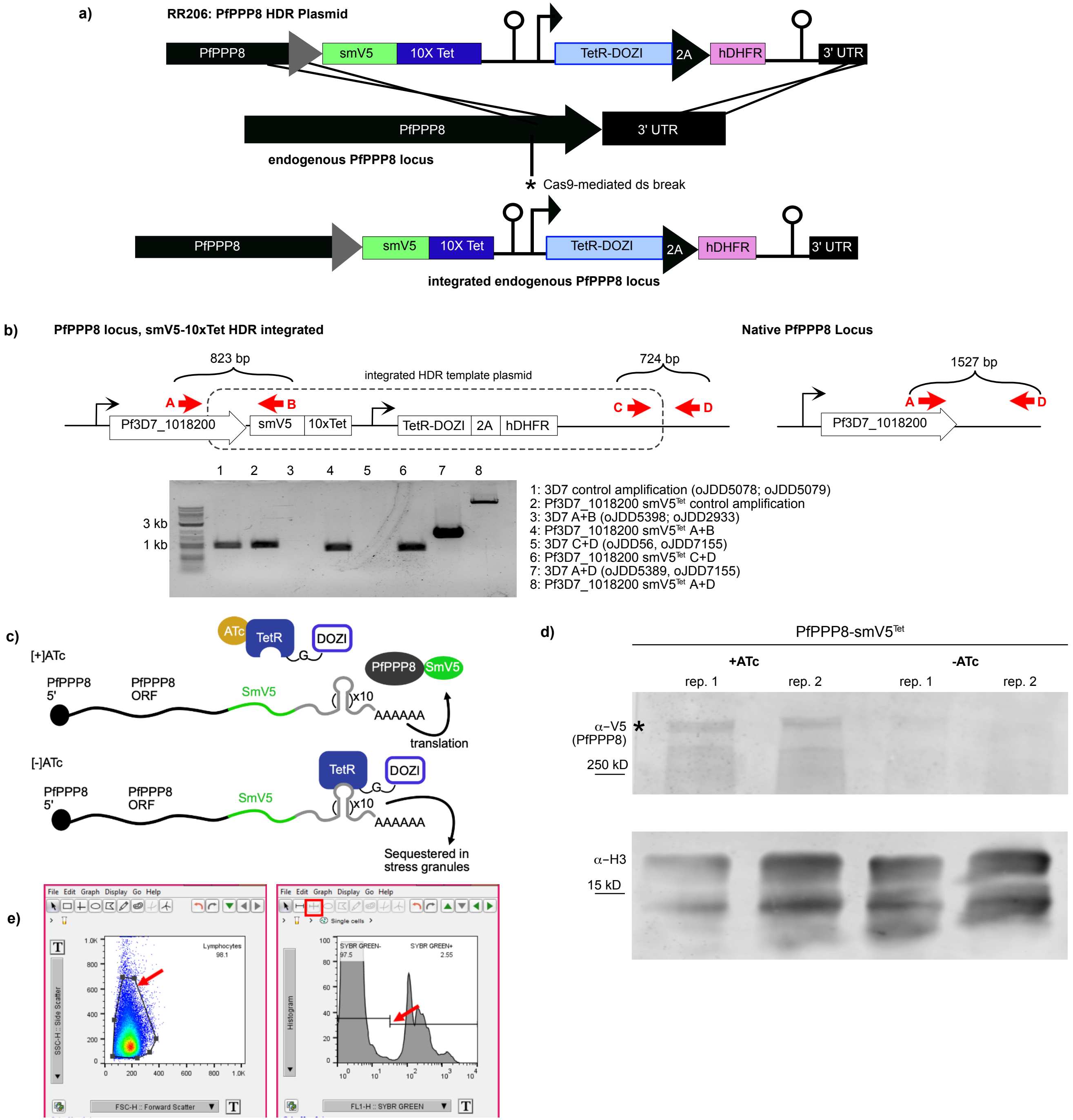

Supplementary Fig. 1 | PfPPP8-smV5^{Tet} targeting construct and knockdown efficiency
a, Schematic of PfPPP8 targeting construct to generate PfPPP8-smV5^{Tet}, the location of its modification, and of the endogenous PfPPP8 locus following integration of construct. **b,** For the PfPPP8 locus in the PfPPP8-smV5^{Tet} line, schematic showing the locus upon HDR integration and the native locus. Below, PCR amplifications of bracketed regions utilizing primers labelled in red; reactions labelled to the right of the image. Amplifications in the modified line are run adjacent to the same reaction in the parental (3D7) line. **c,** Schematic of TetR-DOZI system for translational repression of PfPPP8. **d,** Immunoblot of PfPPP8-smV5^{Tet} protein levels + and - ATc; 2 biological replicates were run on the same gel. Asterisk indicates primary translation product of PfPPP8-smV5^{Tet}. e, gating strategy for flow cytometry data collected for figure 1b. First gating utilized forward and side scatter to identify individual RBCs; this population was then gated based on SYBR Green expression; above 10^1 was considered SYBR Green positive.

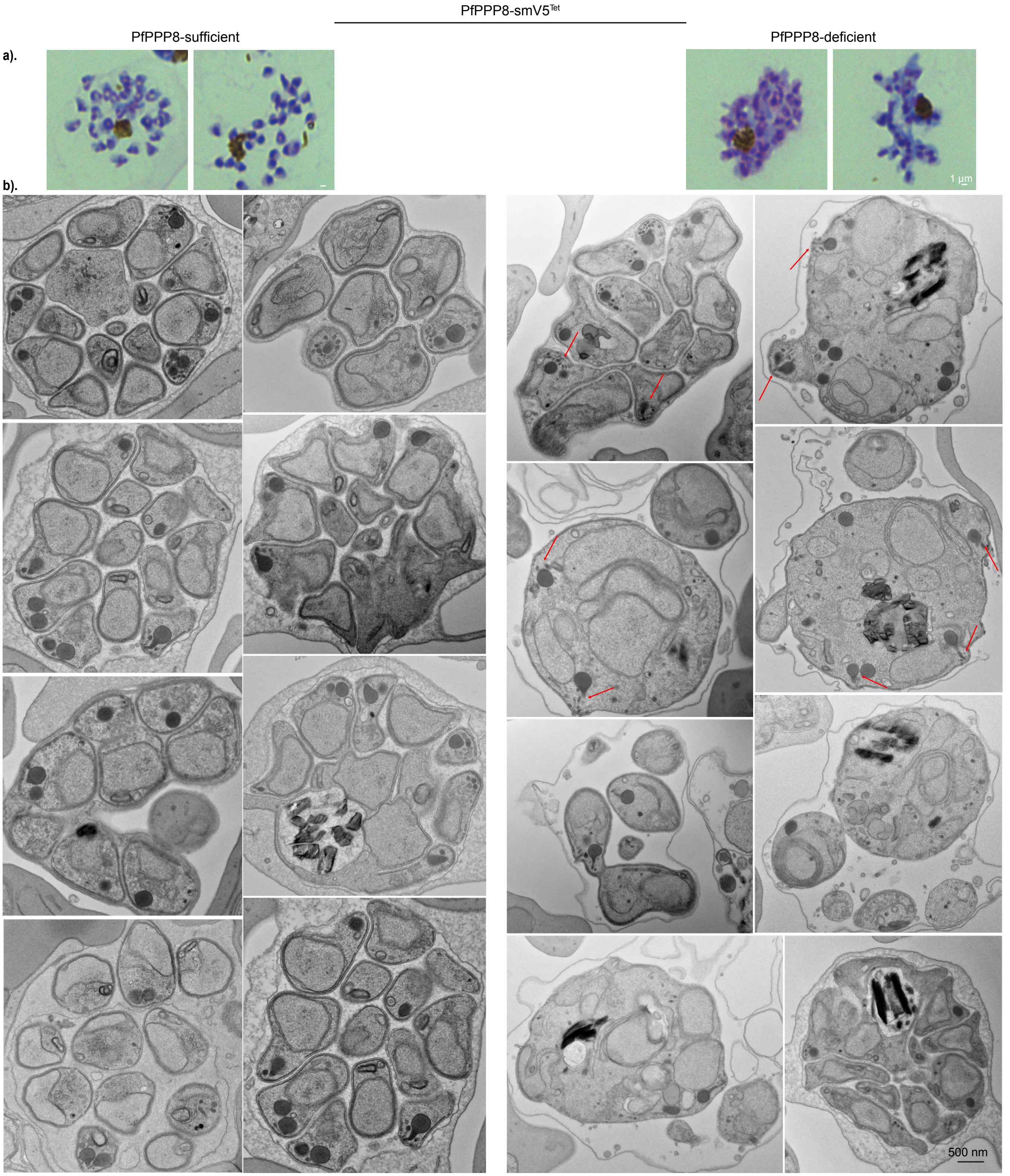

Supplementary Figure 2 | Additional characterization of PfPPP8-deficient phenotype – broad segmentation defects
a, Comparison of merozoite segmentation and individualization in PfPPP8-sufficient (left) and deficient (right) E64-stalled parasites via Field's stain. **b,** Extra examples of PfPPP8-sufficient (left column) compared to -deficient (right column) fully segmented (E64-stalled) parasites via transmission electron microscopy. PfPPP8-deficient parasites display defective segmentation resulting in few merozoites of vastly different sizes, and inability of merozoites to separate from each other indicated by presence of multiple apical ends in a single merozoite (red arrows point to apical organelles in images where more than one set of apical organelles is visible in one merozoite).

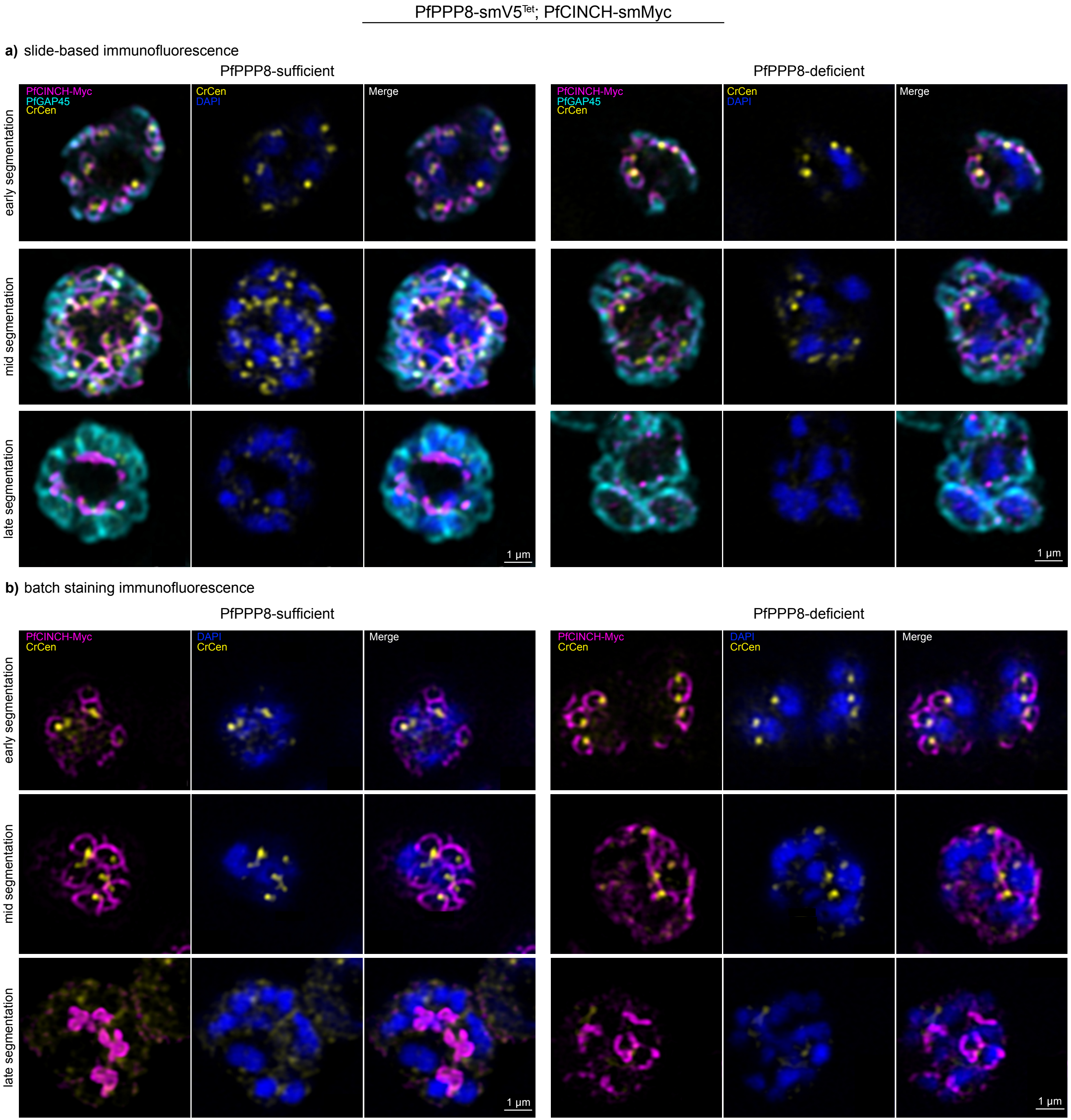

Supplementary Figure 3 | Additional characterization of PfPPP8-deficient phenotype – centrin localization and phenotype
a, Comparison of centrin phenotypes (CrCen primary antibody, yellow) in relation to the basal complex (PfCINCH-smMyc, magenta) and inner membrane complex (PfGAP45, cyan) in early, mid, and late segmentation PfPPP8-sufficient (left) and -deficient (right) parasites by slide IFA. **b,** Comparison of centrin phenotypes (CrCen primary antibody, yellow) in relation to the basal complex (PfCINCH-smMyc, magenta) in early, mid, and late segmentation PfPPP8-sufficient (left) and -deficient (right) parasites by batch-staining IFA.

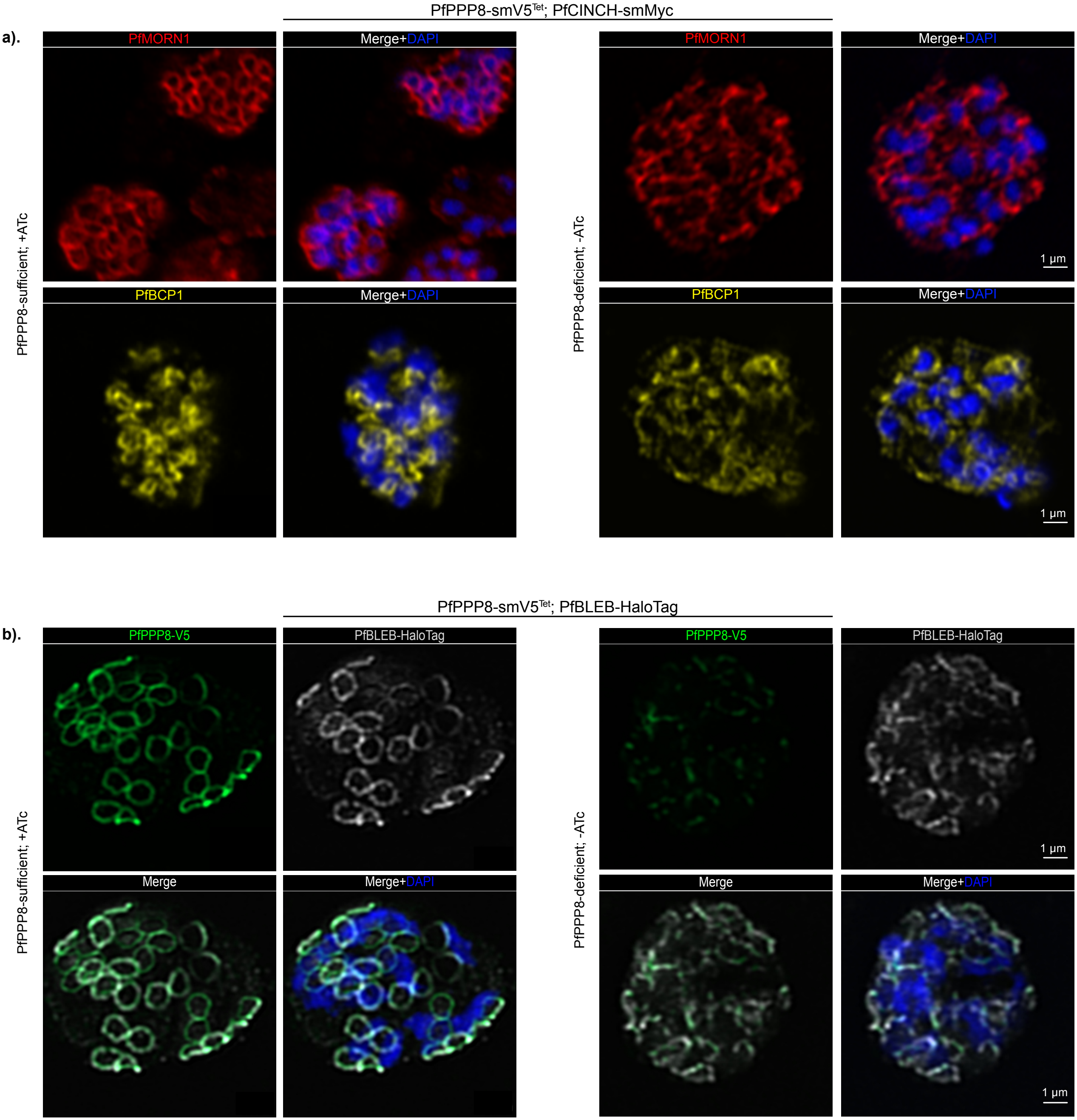

Supplementary Figure 4 | Initial characterization of PfPPP8-deficient basal complex
a, Comparison of basal complex phenotypes using primary antibodies against PfBCP1 (yellow) and PfMORN1 (red) in PfPPP8-sufficient (left) and -deficient (right) parasites by IFA. **b,** Comparison of basal complex phenotype (PfBLEB-HaloTag) in the PfPPP8-smV5^{Tet}-PfBLEB-HaloTag line.

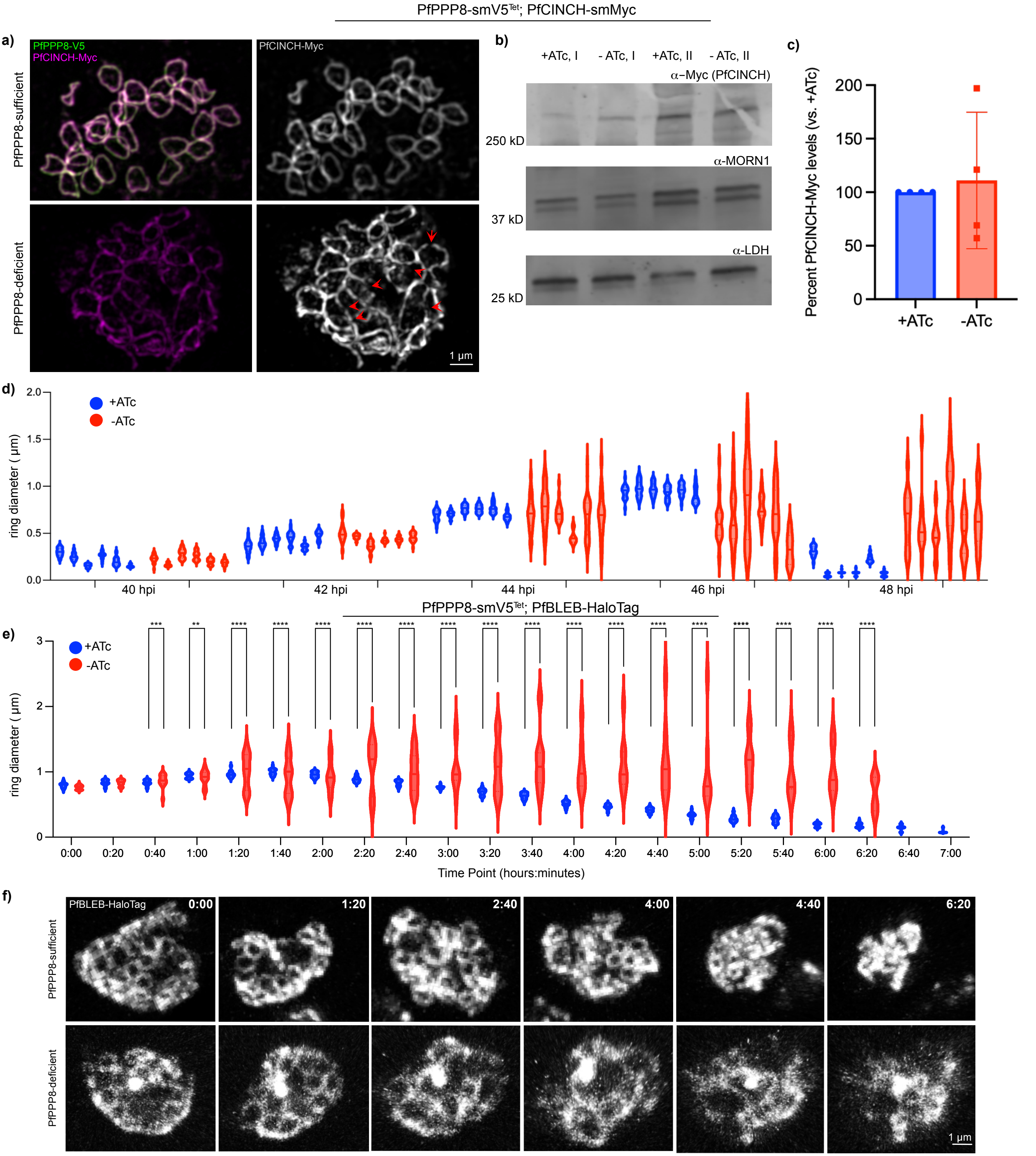

Supplementary Fig. 5 | Additional data on PfPPP8-deficient basal complex integrity and stability during segmentation
a, Comparison of basal complex phenotype (PfCINCH-smMyc) in PfPPP8-sufficient and -deficient parasites by IFA using 3D-Structured Illumination Microscopy. Red arrows point to breakage points in the basal complex. **b,** Immunoblot of basal complex protein levels (PfCINCH-smMyc and PfMORN1) in PfPPP8-sufficient and PfPPP8-deficient parasites; two replicates were run on the same gel. **c,** Quantification of relative PfCINCH protein levels in PfPPP8-deficient compared to PfPPP8-sufficient conditions (data presented as mean values ± SD; n=4 biologically independent samples). **d,** Visualization of 6 individual parasites' basal complex diameter measurements of each condition and time point from Fig. 3b data (n=10-30 rings each). **e,** Quantification of basal complex 'ring' diameter in a single PfPPP8-deficient and PfPPP8-sufficient parasite over the course of imaging; one-sided F-test for equality of variances (0:40: p=0.0006; 1:00: p=0.0013; 1:20-onward: p<0.0001, n=10-25 BC rings). Time point = time after initiation of imaging. **f,** Selected time points (time represented as hours:minutes on each image) showing basal complex phenotype (PfBLEB-HaloTag) in the same parasite (PfPPP8-sufficent and -deficient) over time (see Supplementary Videos 5, 6).

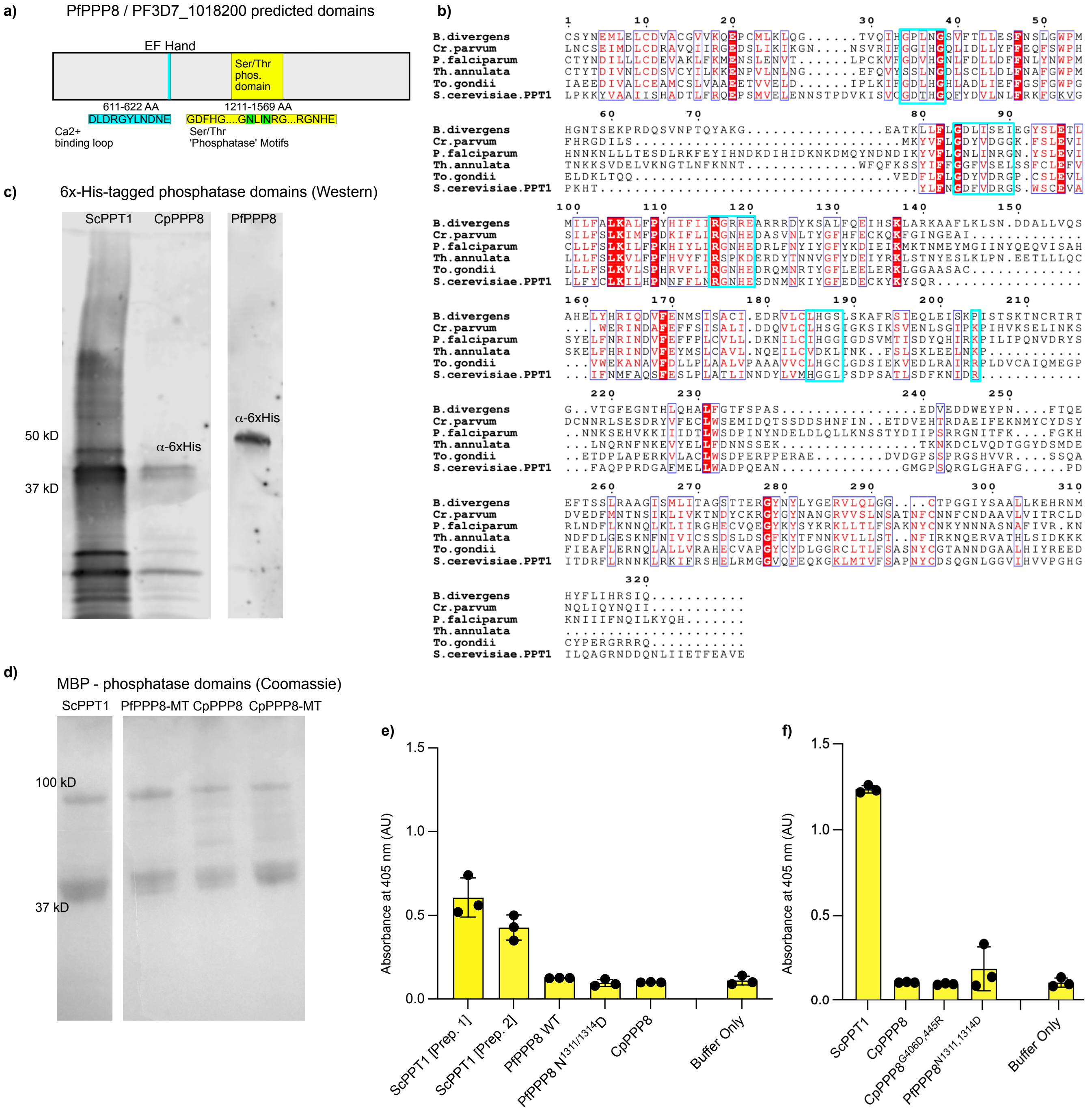

Supplementary Fig. 6 | Additional data on PfPPP8 and homologs' conserved sequences and phosphatase activity of recombinant proteins. **a,** Schematic of domains identified bioinformatically in PfPPP8. **b,** Sequence alignment generated by MegaX, prepared for publication using ESPript3 of predicted phosphatase domains of PfPPP8 and homologs (*T. annulata*, *C. parvum*, *B. divergens, and T. gondii*) plus serine-threonine protein phosphatase *S. cerevisiae* PPT1. Catalytically important motifs boxed in cyan. **c,** Immunoblot of recombinant 6xHis-tagged phosphatase domains of PPT1, PfPPP8, and *C. parvum* PPP8. **d,** Coomassie staining of recombinant maltose-binding protein (MBP)-tagged PfPPP8^{N1311,1314D} , CpPPP8, CpPPP8^{G406D,445R}, and ScPPT1. Expected size of fusion protein for all ~90 kD; of *E. coli* MBP ~44 kD. **e,** pNPP dephosphorylation assay for 6xHis-recombinant phosphatase domains. **f,** pNPP dephosphorylation assay for MBP-recombinant phosphatase domains (for **e** and **f**, n=3 samples run in parallel, representative of multiple biological replicates; data presented as mean values ± SD).

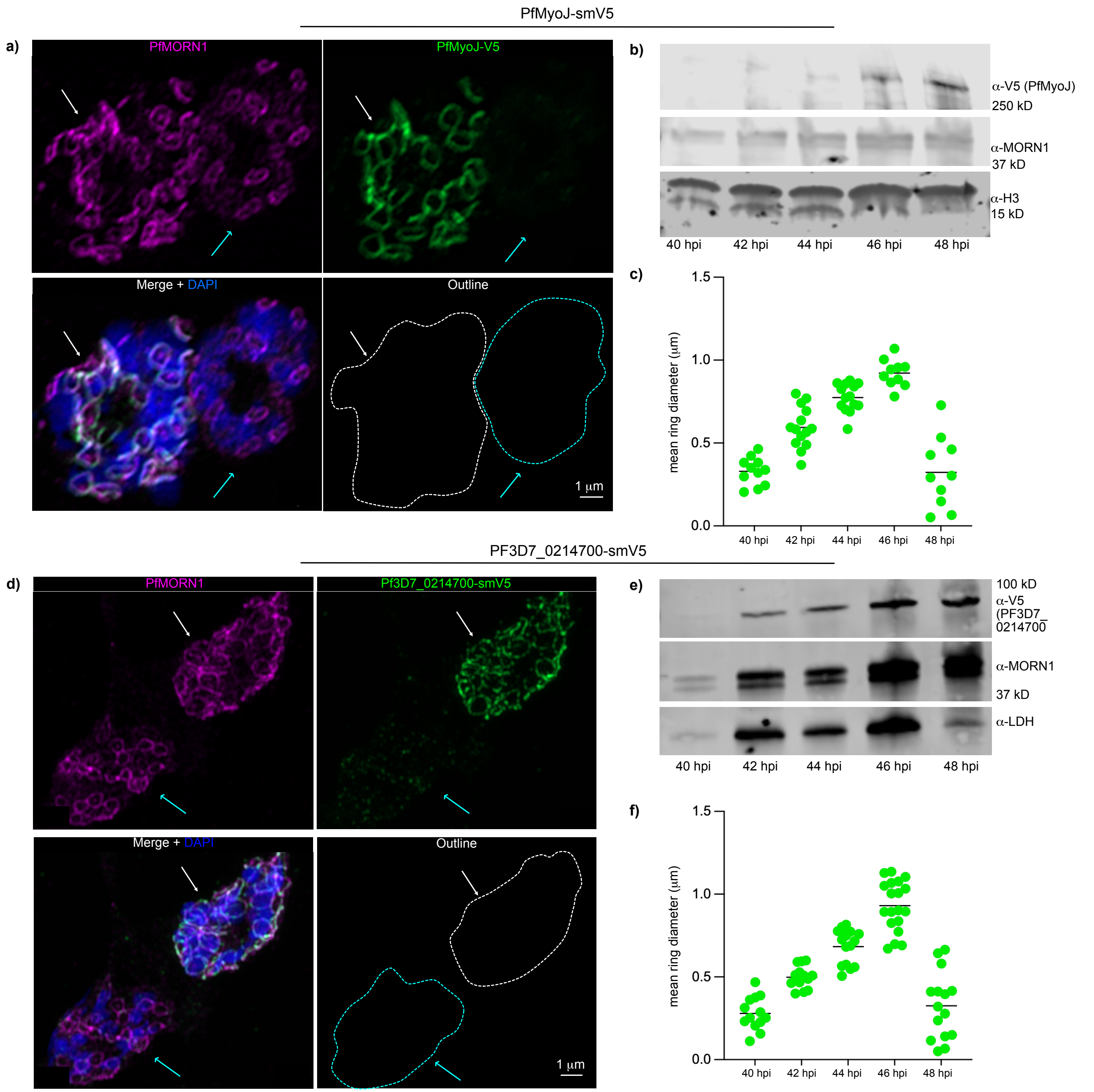

Supplementary Figure 7 | Additional data on PfMyoJ and PF3D7_0214700 temporal localizations
a, Comparison of PfMyoJ and PfMORN1 temporal localization in adjacent earlier and later schizonts of a mixed population by IFA. Earlier schizont indicated with a cyan arrow and a cyan-dashed outline in the fourth panel (defining the boundaries of each parasite). Later schizont indicated with a white arrow and a white-dashed outline in the fourth panel. **b,** Immunoblot comparing PfMyoJ and PfMORN1 protein levels in a tightly synchronized population, with samples taken every 2 hours. **c,** Quantification of mean BC ring diameter at each time point represented in b; n= 10 schizonts per time point. **d,** Comparison of PF3D7_0214700 and PfMORN1 temporal localization in adjacent earlier and later schizonts of a mixed population by IFA. Earlier schizont indicated with a cyan arrow and a cyan-dashed outline in the fourth panel (defining the boundaries of each parasite). Later schizont indicated with a white arrow and a white-dashed outline in the fourth panel. **e,** Immunoblot comparing PF3D7_0214700 and PfMORN1 protein levels in a tightly synchronized population, with samples taken every 2 hours. **f,** Quantification of mean BC ring diameter at each time point represented in **e**; n= 10 schizonts per time point.

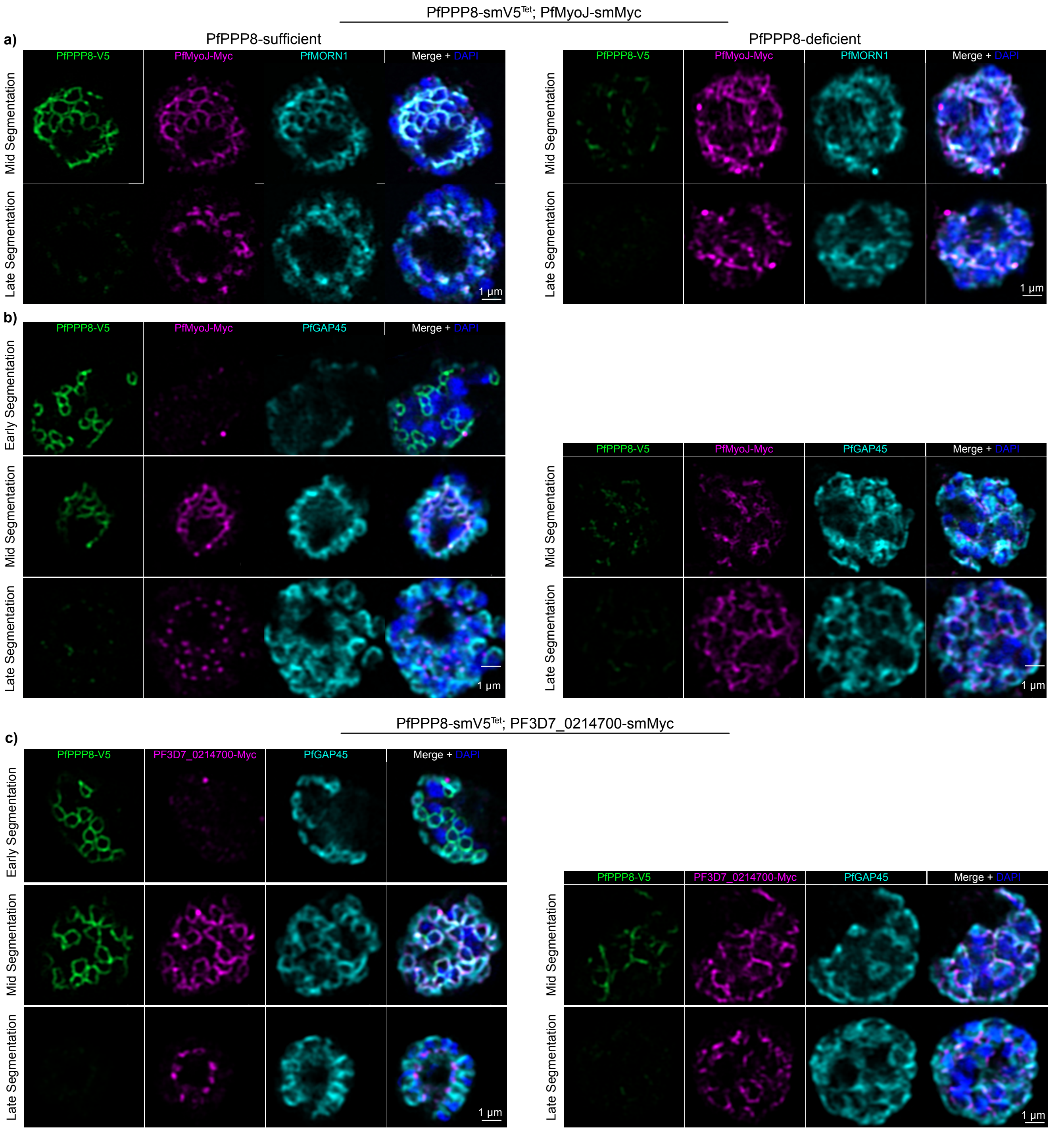

Supplementary Figure 8 | PfMyoJ and PF3D7_0214700 are recruited to the basal complex even in PfPPP8-deficient parasites.
a, Comparison of PfMyoJ (PfMyoJ-smMyc) and PfMORN1 phenotypes in PfPPP8-sufficient (left) and -deficient (right) parasites by IFA, using the PfPPP8-smV5^{Tet}; PfMyoJ-smMyc line. **b,** Comparison of PfMyoJ (PfMyoJ-smMyc) and PfGAP45 phenotypes in PfPPP8-sufficient (left) and -deficient (right) parasites by IFA, using the PfPPP8-smV5^{Tet}; PfMyoJ-smMyc line. **c,** Comparison of PF3D7_0214700 (PF3D7_0214700-smMyc) and PfGAP45 phenotypes in PfPPP8-sufficient (left) and -deficient (right) parasites by IFA, using the PfPPP8-smV5^{Tet}; PF3D7_0214700-smMyc line.

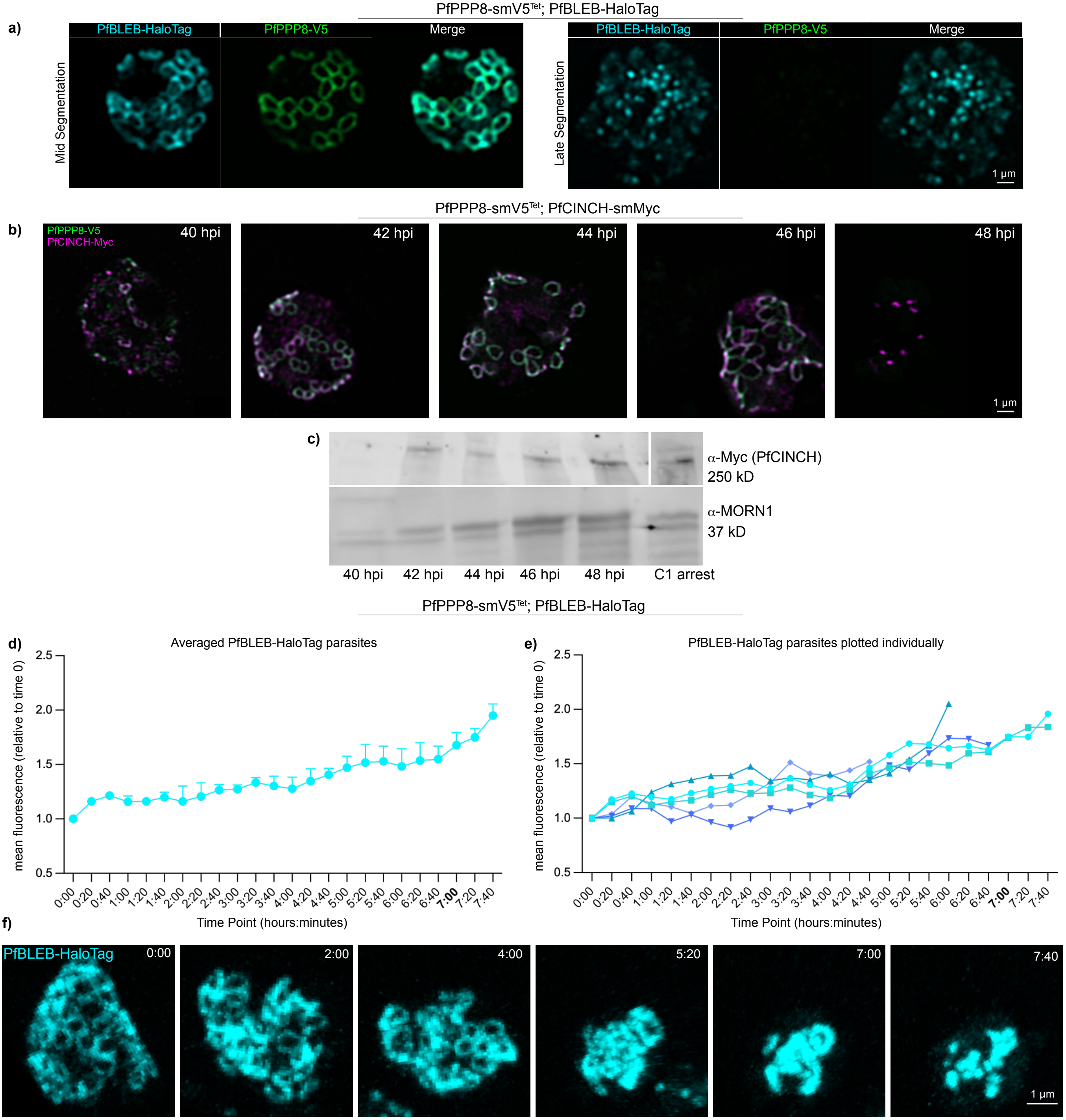

Supplementary Figure 9 | Additional data comparing PfPPP8's temporal localization to PfBLEB, PfCINCH, and PfMORN1
a, Comparison of PfPPP8-smV5 and PfBLEB-HaloTag expression in PfPPP8-smV5^{Tet}; PfBLEB-HaloTag parasites at the middle and end of segmentation. **b,** Regular IFA timeline of PfPPP8 and PfCINCH expression from 40 to 48 hours with images taken every 2 hours in PfPPP8-smV5^{Tet}; PfCINCH-smMyc parasites. **c,** Immunoblot comparing PfCINCH-smMyc and PfMORN1 expression over the course of segmentation, from fig. 6b samples. **d,** Quantification of relative PfBLEB-HaloTag fluorescence, compared to fluorescence at the onset of imaging, for averaged PfPPP8-smV5^{Tet}; PfBLEB-HaloTag parasites (n=5 parasites, matched by initial BCD) (time represented as hours:minutes on each image. Data presented as mean values ± SD). **e**, Quantification of relative fluorescence, compared to fluorescence at the onset of imaging, for individual PfBLEB-HaloTag parasites in **d**. Time point = time after initiation of imaging. **f,** Selected time points (time represented as hours:minutes on each image) demonstrating PfBLEB-HaloTag fluorescence in one of the schizonts analyzed in **d** & **e**.

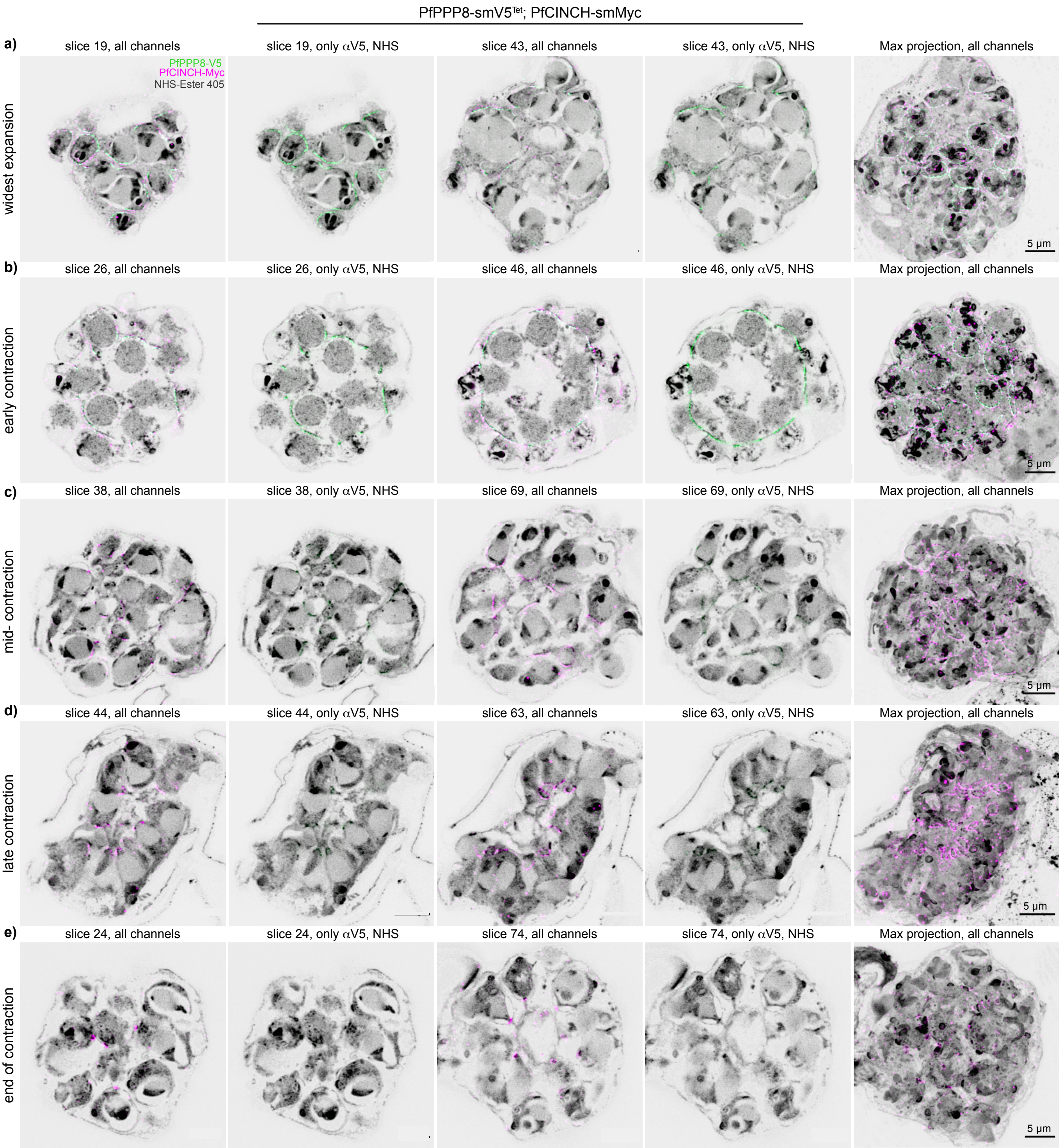

Supplementary Fig. 10 | Expansion microscopy shows PfPPP8-depletion over the course of basal complex contraction.
a, Individual slices from a mid-segmentation schizont with both tagged proteins or only PfPPP8-V5 displayed. Maximum projection demonstrates ring size and shape. **b,** Individual slices from a schizont beginning basal complex contraction with both tagged proteins or only PfPPP8-V5 displayed. Maximum projection demonstrates ring size and shape. **c,** Individual slices from a schizont midway through basal complex contraction with both tagged proteins or only PfPPP8-V5 displayed. Maximum projection demonstrates ring size and shape. d, Individual slices from a schizont finishing basal complex contraction with both tagged proteins or only PfPPP8-V5 displayed. Maximum projection demonstrates ring size and shape. **e,** Individual slices from a schizont having completed basal complex contraction with both tagged proteins or only PfPPP8-V5 displayed. Maximum projection demonstrates ring size and shape.

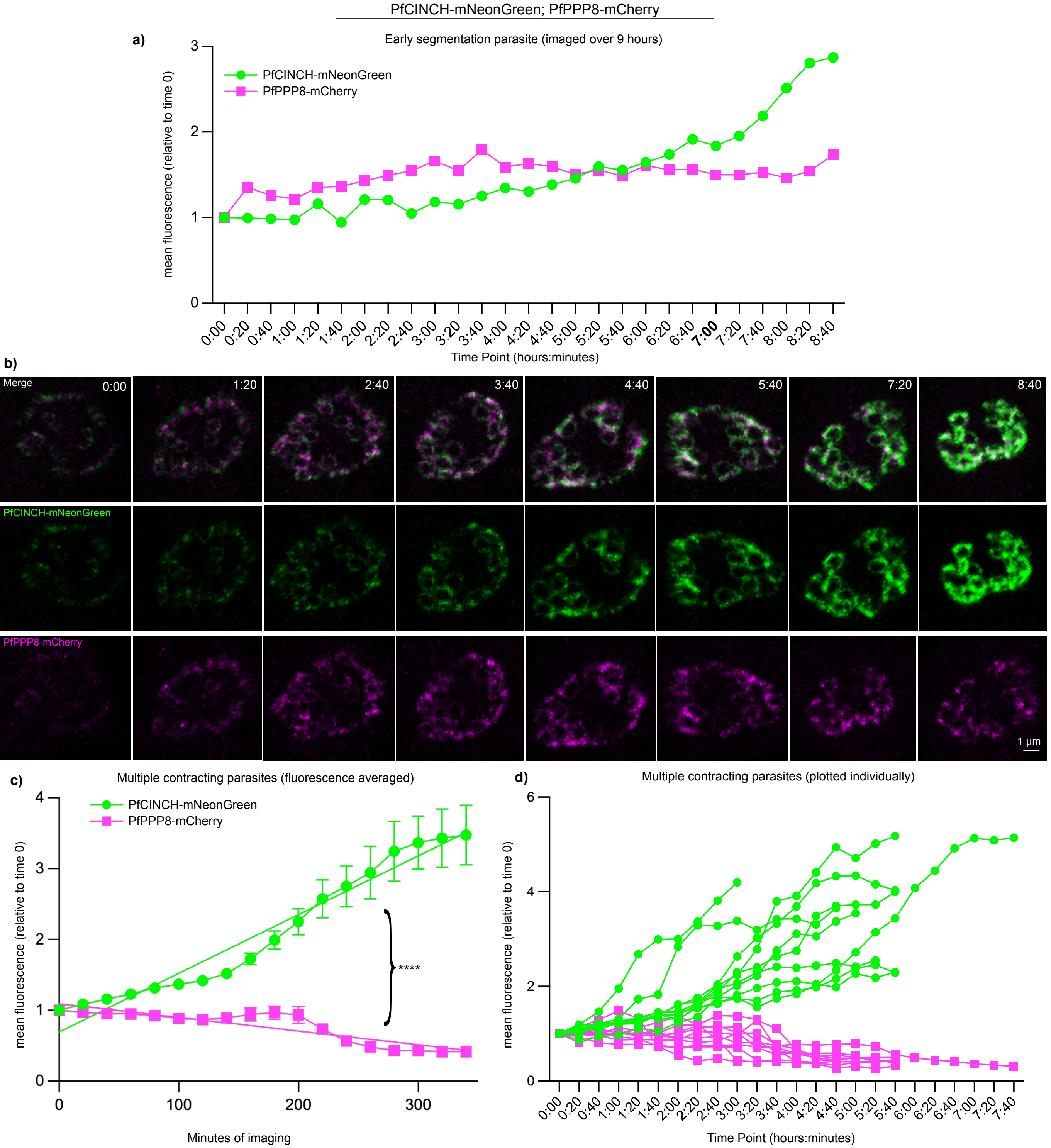

Supplementary Fig. 11 | Additional data on PfPPP8's temporal localization in the PfCINCH-mNeonGreen; PfPPP8-mCherry parasites.
a, Quantification of relative fluorescence, compared to fluorescence at the onset of imaging, for both PfCINCH-mNeonGreen and PfPPP8-mCherry in an early segmentation schizont. Time point = time after initiation of imaging. **b,** Selected time points (time represented as hours:minutes on each image) from Supplementary Video 9, corresponding to time points in a, comparing PfCINCH-mNeonGreen and PfPPP8-mCherry fluorescence. **c,** Quantification of relative fluorescence, compared to fluorescence at the onset of imaging, for averaged PfCINCH-mNeonGreen and PfPPP8-mCherry parasites (n=7 parasites, representing multiple biological replicates; data presented as mean values ± SD). Regression analysis created a line of best fit for each protein's fluorescence and the slopes are significantly different (two-tailed t test; $p<0.0001$; Analysis of Covariance). **d,** Quantification of relative fluorescence, compared to fluorescence at the onset of imaging, for individual PfCINCH-mNeonGreen; PfPPP8-mCherry parasites in **c**. Time point = time after initiation of imaging

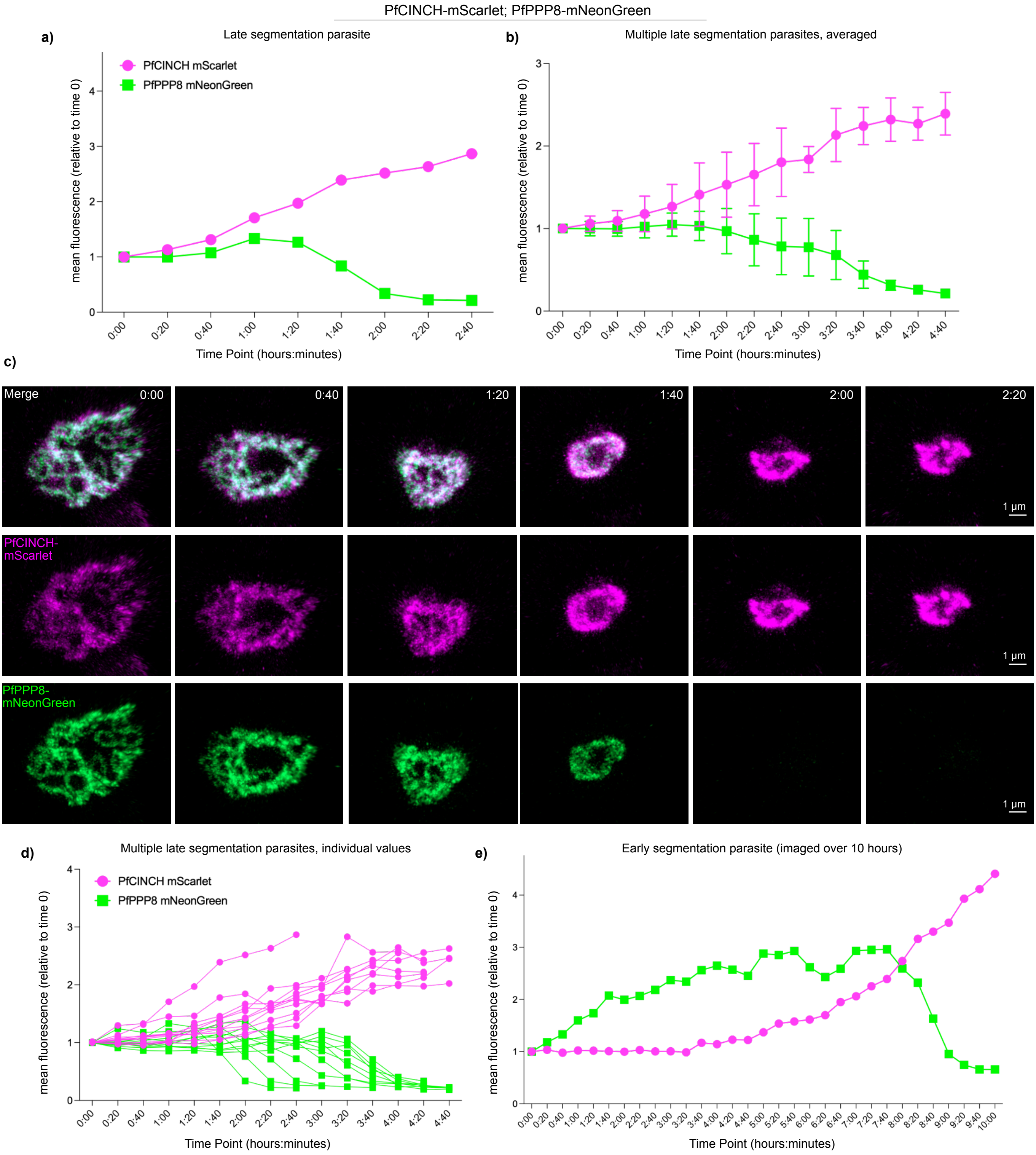

Supplementary Figure 12 | Data on PfPPP8's temporal localization in PfCINCH-mScarlet; PfPPP8-mNeonGreen parasites.
a, Quantification of relative fluorescence, compared to fluorescence at the onset of imaging, for both PfCINCH-mScarlet and PfPPP8-mNeonGreen in a late segmentation schizont. **b,** Quantification of relative fluorescence, compared to fluorescence at the onset of imaging, for averaged PfCINCH-mScarlet and PfPPP8-mNeonGreen parasites (n=10 parasites, representing multiple biological replicates; data presented as mean values ± SD). **c,** Selected time points (time represented as hours:minutes on each image) from Supplementary Video 10, corresponding to time points in **a,** comparing PfCINCH-mScarlet and PfPPP8-mNeonGreen fluorescence. **d,** Quantification of relative fluorescence, compared to fluorescence at the onset of imaging, for individual PfCINCH-mScarlet; PfPPP8-mNeonGreen parasites in b. **e,** Quantification of relative fluorescence, compared to fluorescence at the onset of imaging, for both PfCINCH-mScarlet and PfPPP8-mNeonGreen in an early segmentation schizont (see Supplementary Video 11).

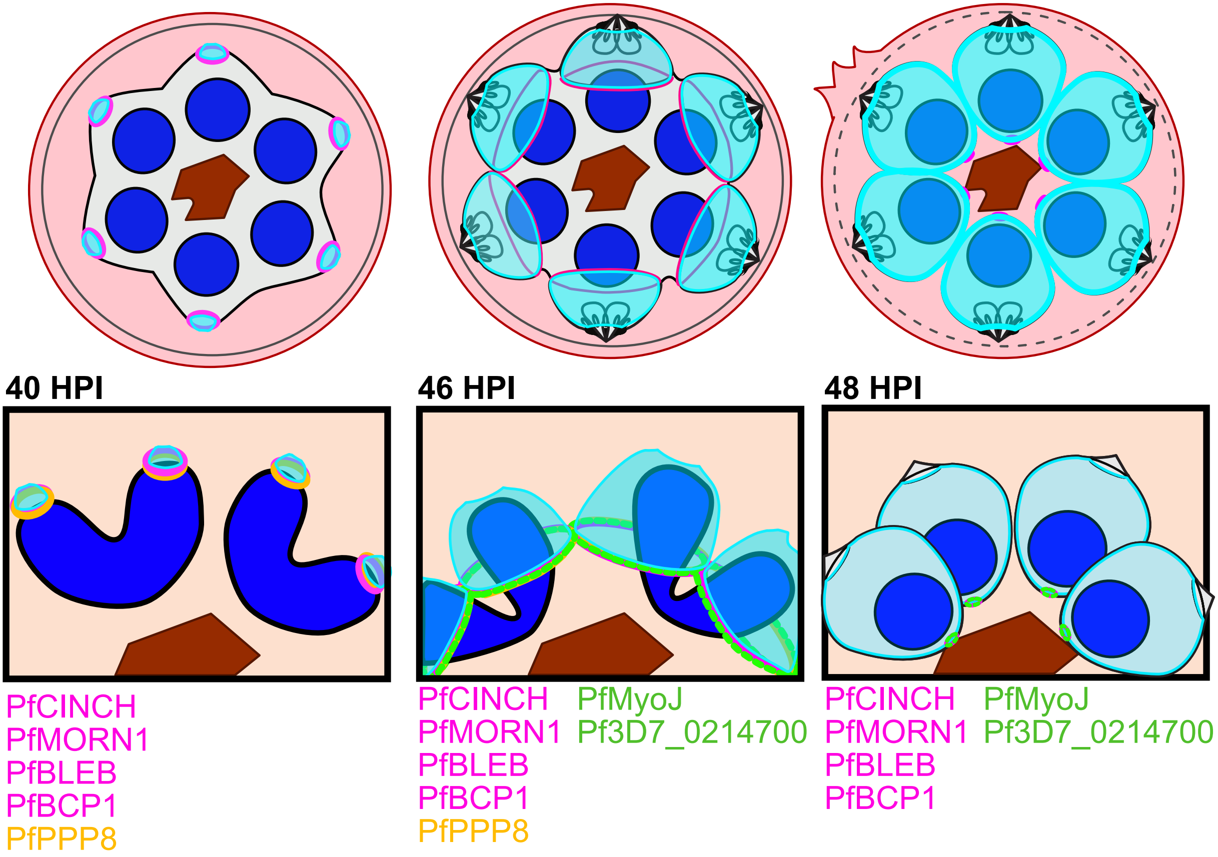

Supplementary Figure 13 | Basal Complex Timing Model
Top row: simplified model of the process of segmentation in *Plasmodium* schizonts dividing in the RBC, with three time points depicted. The nucleus is dark blue, inner membrane complex cyan, basal complex magenta, hemozoin crystal red-orange. Bottom row: 'zoomed-in' representations of the basal complex in more detail at each stage, with selected BC proteins colored according to their temporal localization. In magenta, the bulk of the known basal complex proteins which are present throughout segmentation. In orange, proteins which depart before segmentation ends but are present early in the process. In green, proteins which are recruited partway through segmentation. This model is meant only to demonstrate temporal relationships between basal complex proteins; spatial relationships of proteins to each other within the ring should not be inferred from the arrangement of colors in the diagram

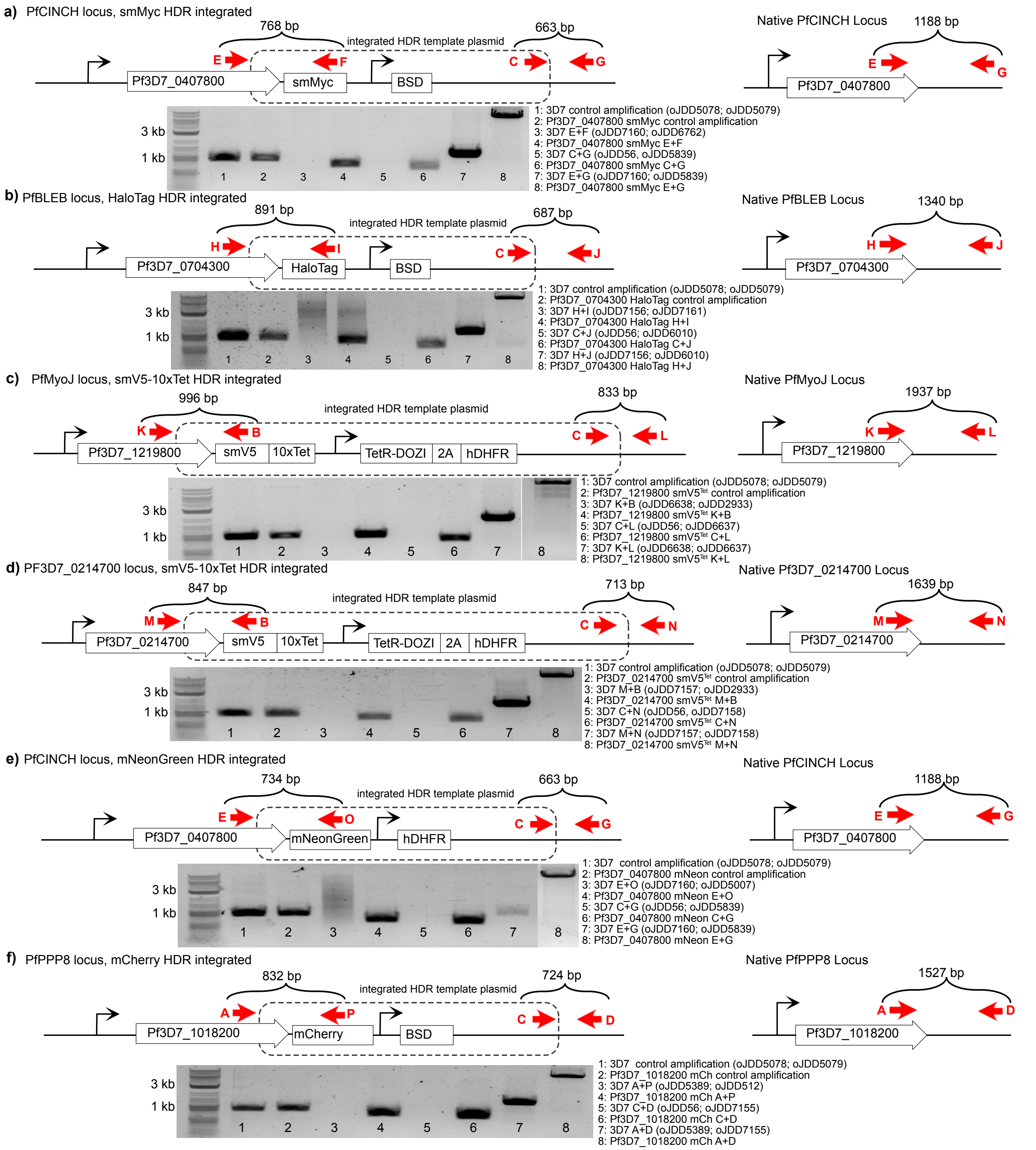

Supplementary Fig. 14 | Integration PCRs for 5 of the 8 lines generated and utilized.
a, For the PfCINCH locus in the PfPPP8-smV5^{Tet}; PfCINCH-smMyc line, schematic showing the locus upon HDR integration and the native locus. Below, PCR amplifications of bracketed regions utilizing primers labelled in red; reactions labelled to the right of the image. Amplifications in the modified line are run adjacent to the same reaction in the parental (3D7) line. **b – f** are identical in setup, providing the same information about each genetically modified parasite generated and utilized.

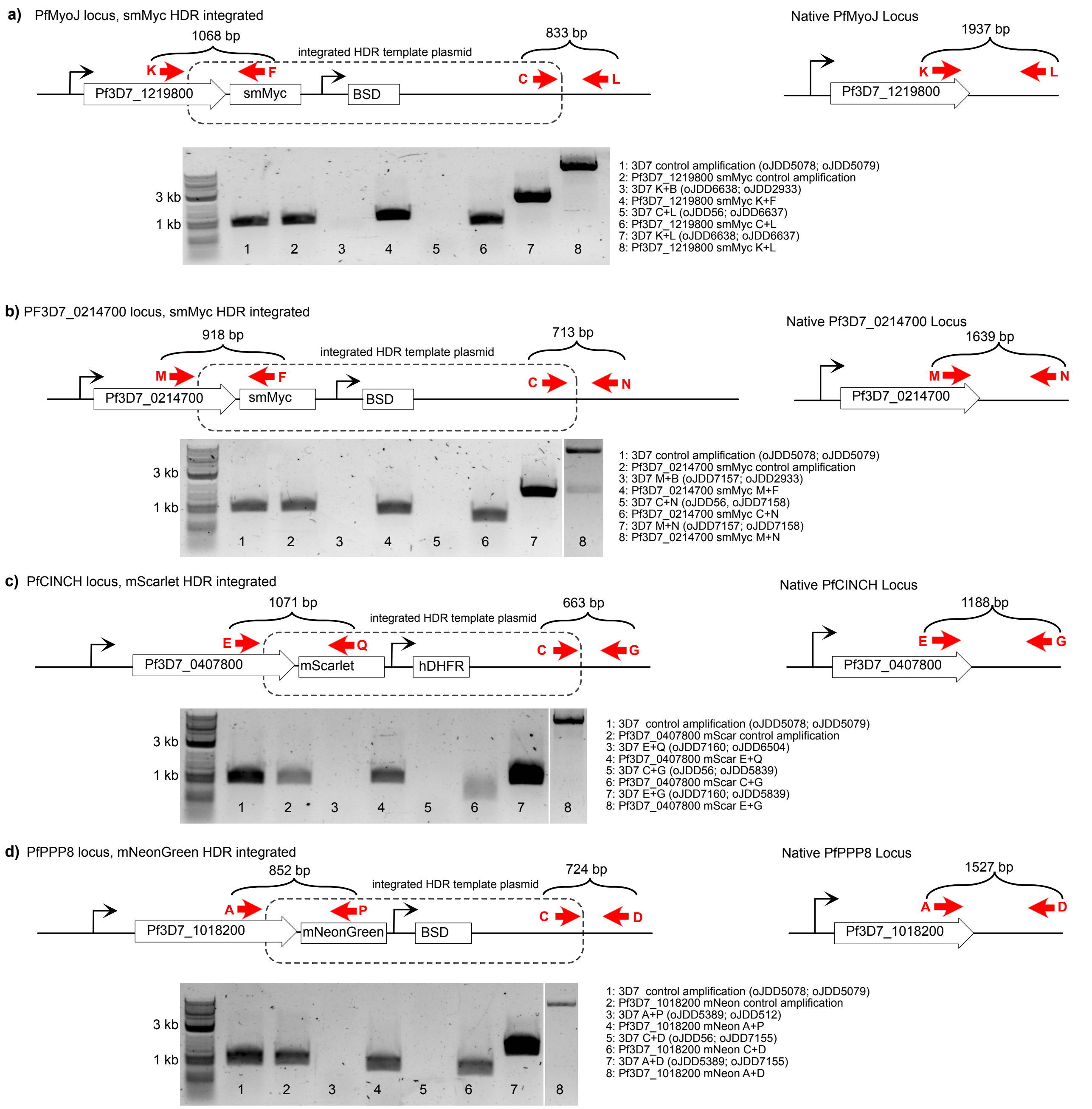

Supplementary Fig. 15 | Integration PCRs for 3 of the 8 lines generated and utilized.
a, For the PfPPP8-smV5^{Tet}; PfMyoJ-smMyc line, schematic showing the locus upon HDR integration and the native locus. Below, PCR amplifications of bracketed regions utilizing primers labelled in red; reactions labelled to the right of the image. Amplifications in the modified line are run adjacent to the same reaction in the parental (3D7) line. **b – d** are identical in setup, providing the same information about each genetically modified parasite generated and utilized.

Supplementary Note 1

```
                     1         10            20                      30         40         50
B.sp.xinjiang        CSYNELLELCDVVSNIVKQETTVVN.LQGT......TQLHGPLNGNVLTLLESFNSLGWP
B.bigemina           CSYNELLELCDVVCGVVRQEGSVLH.MEGT......VQMHGPLNGSVLTLLESFNCLGWP
B.ovata              CSYNELLELCDVVCGVVRQESSVLY.LQGT......VQVHGPLNGSVLTLLESFNCLGWP
B.divergens          CSYNEMLELCDVACGVVKQEPCMLK.LQGT......VQIHGPLNGSVFTLLESFNSLGWP
B.bovis              CSYNELVELCDVMCHIVRKEGALVR.LQGT......TQLHGPLNGDVYTLLDTFNSLGWP
B.microti            CNYHSLLVLCNNFSHLLAKEDTIVK.IKSP......TRIIGELGNNFVDLIRTFNIYGWP
Be.besnoiti          LALEDMVALFDACSVLFAAEETVIE.VNL......PSKIFGDIHGHLADLLEFFGSFGWP
Ch.velia             CTAEEMLCLCDRVVLLFMEEDTLVD.VLL......PCRVYGDVHGQLPDLLALFETFSWP
Cr.muris             ATVDEIMKLCDTVVELFRKENSLIQ.NSEF...KMPVRIFGDIHGQLFDILHFFEKFSWP
Cr.andersoni         ATVDEIMKLCDTVVELFRKENSLIQ.NSEF...KMPVRIFGDIHGQLFDILHFFEKFSWP
Cr.ubiquitum         LSYSEIMDLCDRLVQIIRNENSLVR.IKA....NNSVRIFGGIHGQLIDLLYFFEHFSWP
Cr.meleagridis       LSCSEIMDLCDRAVQIIRGENSLIK.IKR....NNSVRIFGGIHGQLIDLLYFFEQFSWP
Cr.hominis           LNCSEIMDLCDRAVQIIRGEDSLIK.IKG....NNSVRIFGGIHGQLIDLLYFFEQFSWP
Cr.parvum            LNCSEIMDLCDRAVQIIRGEDSLIK.IKG....NNSVRIFGGIHGQLIDLLYFFEQFSWP
Cr.tyzzeri           LDCSEIMDLCDRAVQIIRGEDSLIK.IKG....NNSVRIFGGIHGQLIDLLYFFEQFSWP
Cr.sp.chipmunk       LNYSEIMDLCDRAVQIIRSEDSLLT.IKG....NSSVRIFGGIHGQLIDLLYFFENFSWP
Cr.ryanae            LNHLEIIELCDQVVEVIRNENSLIR.INKE...NNAIRIFGGLYGQLSDLLSLFESFSWP
Cr.bovis             LNYLEIMKLCDQVVEVIRDENSLIR.LNGE...NNTIRIFGGLYGQLSDLLHLFENFSWP
Cyc.cayetanensis     ATASEALLLCDAVASILALENSLLE.LEL......PLRVYGDIHGQLGDLLDFFDSFLWP
Cys.suis             ATPDEILTILEAVKCIFSVETTVLE.VNL......PAKIFGDLHGHLADLLEFFRSFGWP
Cyt.felis            CTYNEMINLFDTVYTLIRREDSLIY.IDGI.....VQIHGDMNNDLYSLIDIFNTYGWP
E.tenella            ATAEEALKLCDATASLVAAENSLLE.LEL......PLRVYGDLHGQLVDLLDFFGSFGWP
E.falciformis        ATAQEALTLCDAVASLVAREDSLLE.LEL......PLRVYGDLHGQLGDLLDFFNSFGWP
H.hammondi           VAEEDIVALCDAVGSLVAAEETVIE.LDI......PLKIFGDLHGHLADLIEFFGSFGWP
N.caninum            LAVEEVLALCDAVERLVAAEETVIE.LDV......PLKIYGDLHGHLADLLEFFGSFGWP
P.falciparum         CTYNDILLLCDEVAKLFKMENSLEN.VTLP.....CKVFGDVHGNLFDLLDFFNLYNWP
P.praefalciparum     CTYNDILLLCDEVAKLFKMENSLEN.VTLP.....CKVFGDVHGNLFDLLDFFNLYNWP
P.reichenowi         CTYNDILLLCDEVAKLFKMENSLEN.VTLP.....CKVFGDVHGNLYDLLDFFNLYNWP
P.blacklocki         CTYNDILLLCDEVAKLFKIENSLEN.VTLP.....CKVFGDVHGNLYDILDFFNLYNWP
P.billcollinsi       CTYNDILLLCDEVAKLFKIENSLEN.VTLP.....CKVFGDIHGNLYDILDFFNLYNWP
P.adleri             CTYNDILLLCDEVAKLFKIENSLEENVSLP.....CKVFGDIHGNLYDILDFFNLYNWP
P.gaboni             CTYNDILLLCDEVAKLFKIENSLEENVSLP.....CKVFGDVHGNLYDILDFFNLYNWP
P.gallinaceum        CSYNDILLLCDEVAKLFKLENSLEN.VKLP.....CKVFGDIHGNLFDIMDFFNLYNWP
P.vivax              CSYNDVILLCDEAAKVLKGEDSIEQ.VELP.....CKVFGDLHGNVLDLLDFFNMYGWP
P.fragile            CTYNDIILLCDEAVKVFKGEDSIEH.VDLP.....CKVFGDLHGNIFDLLDFFNMYNWP
P.knowlesi           CTYNDIILLCDEAVKVFKGEDTIEY.VDLP.....CKVFGDLHGSIFDLLDFFNMYNWP
P.coatneyi           CTYNDILLLCDEAVKVFKGEDSIEK.VDLP.....CKVFGDLHGNIFDLLDFFNMYNWP
Th.parva             CTYSDIVNLCDSACYILKKENSLLN.LNGE.....FQVYSSLNGDLSCLLDLFNAYGWP
Th.annulata          CTYTDIVNLCDSVCYILKKENPVLN.LNGE.....FQVYSSLNGDLSCLLDLFNAFGWP
Th.orientalis        CAYSDMLQLCDAACSILKKEDSLLQ.LKGN.....VQVFGSLNGDLSCLLDQFNALDG.
Th.equi              CTYTDLLKLCDSTCNLLKKEDSLLK.LNGS.....VQIYGSINADIISLGKMFNTYGWP
To.gondii            IAEEDIVALCEAMCSLVAAEETVVE.LDI......PLKIFGDLHGHLADLIEFFGSFGWP
V.brassicaformis     CTSDEVKMLCDMVVPHLIHENTLVE.LSL......PVRVYGDIHGNLPDLLTFFNTYAWP
S.cerevisiae.PPT1    LPKKYVAAIISHADTLFRQEPSMVE.LENNSTPDVKISVCGDTHGQFYDVLNLFRKFGKV
```

```
                             60
B.sp.xinjiang        LHGESCDS....................................................
B.bigemina           LHGNTADR....................................................
B.ovata              LHGNTTER....................................................
B.divergens          MHGNTSEK....................................................
B.bovis              LHGDTCDM....................................................
B.microti            LHSRANDDSTALS...............................................
Be.besnoiti          GDIDKITQQME.................................................
Ch.velia             .DKRRGDILSM.................................................
Cr.muris             .HYHRGDILSM.................................................
Cr.andersoni         .HYHRGDILSM.................................................
Cr.ubiquitum         .HFHRGDILSM.................................................
Cr.meleagridis       .HFHKGDILSM.................................................
Cr.hominis           .HFHRGDILSM.................................................
Cr.parvum            .HFHRGDILSM.................................................
Cr.tyzzeri           .HFHRGDILSM.................................................
Cr.sp.chipmunk       .HFHRGDILSM.................................................
Cr.ryanae            .HYQKGDILSM.................................................
Cr.bovis             .HYQKGDILSM.................................................
Cyc.cayetanensis     ..EEAMQQLNT.................................................
Cys.suis             DEVDKTTRQYE.................................................
Cyt.felis            LHN.........................................................
E.tenella            ..EDLISGLGC.................................................
E.falciformis        ..QETIEGLSS.................................................
H.hammondi           DEPDKLTDQVE.................................................
N.caninum            GDEDKLTEQIE.................................................
P.falciparum         MHNNKNLLLTESDLRKFEYIHN...DKDIHIDKNKD.....................MQY
P.praefalciparum     MHNNKNLLLTETDLRKFEYIHN...DKDIHIDKNKD.....................MQY
P.reichenowi         MHNNKNLLLTETDLRKFEYIHN...DKDIHIDKNKD.....................MQY
P.blacklocki         MHNNKNLLLTEMDLRRFEYINN...DKDIHIDKYKD.....................IQY
P.billcollinsi       MHNNKNLLLTERDLIKFEYINN...DKD..IDKKND.....................MQY
P.adleri             MHNNKNLLLTETDLRKFEYMNN...DKNINVDKKND.....................IQY
P.gaboni             MHNNKNLLLTETDLRKFEYINN...HKNINVDKKND.....................IQY
P.gallinaceum        MHNSMNELISINDLINFENMNYNKMNENYSCEKIRDE...................NEEIY
P.vivax              LHGGTNEWLSVEEVAMRG.......GRSVSVGKLKEAVPHTGGTSVSVEKRRETVPHTDG
P.fragile            LHGDTNEWLSVDEVATSD.......ATCMSVGKANEA................SVANMAA
P.knowlesi           MHGETNEWLNVEEVSATN.......EVSLRSKNSTD.................PFPYVAS
P.coatneyi           LHGETNEWLTVEEVATTD.......GMSIPMGKPNE.................TLPHMVR
Th.parva             MTNKRCVDELV.................................................
Th.annulata          MTNKKSVDELV.................................................
Th.orientalis        ............................................................
Th.equi              LHQ.........................................................
To.gondii            GELDKLTQQVE.................................................
V.brassicaformis     .DRRRGDILTT.................................................
S.cerevisiae.PPT1    GPKHT.......................................................
```

```
                                  70        80        90        100       110
B.sp.xinjiang          ...GVNKLLDAGMYAKGEATKLIFLGDMIGDV..EGFSLETLMVLFSLKILFPYHVFLL.
B.bigemina             ...SVARAVDASQYAKGETTKLVFLGDLIGEV..EGFSLETLLVLFSLKVLYPYHVFLL.
B.ovata                ...KGARLVDAGQYAKGETTKLVFLGDLIGEA..EGFSLETLLVLFSLKVLFPYHVILL.
B.divergens            ...PRDQSVNPTQYAKGEATKLLFLGDLISEI..EGYSLETLMILFALKALFPYHIFII.
B.bovis                ...ITNKLADAGMYAKGEATKLVFLGNMIGDV..EGFSMETLLVLFSLKILFPYHVFLL.
B.microti              ...................HIIINICGQINPPHAPVYSLECLLLVMSLKVLFPKHITIVG
Be.besnoiti            ....................DFLFLGDYVDR...GHCSLDVLLLLFSLKVLYPNRVFLL.
Ch.velia               ....................NYVFLGDFVDR...GTFSLEVVALMFSLKLLYPRKVFLL.
Cr.muris               ....................NYIFLGDYVDR...GKFSLEVIFLLFSFKILFPNKIMML.
Cr.andersoni           ....................NYIFLGDYVDR...GKFSLEVIFLLFSFKILFPNKIIML.
Cr.ubiquitum           ....................KYVFLGDYVDG...GKFSLEVISILFSLKIMFPDKIFLL.
Cr.meleagridis         ....................KYVFLGDYVDR...GKFSLEVISILFSLKIMFPDKIFLL.
Cr.hominis             ....................KYVFLGDYVDG...GKFSLEVISILFSLKIMFPDKIFLL.
Cr.parvum              ....................KYVFLGDYVDG...GKFSLEVISILFSLKIMFPDKIFLL.
Cr.tyzzeri             ....................KYVFLGDYVDG...GKFSLEVISILFSLKIMFPDKIFLL.
Cr.sp.chipmunk         ....................RYVFLGDYVDG...GRFSLEVISILFSLKIMFPDKIFLL.
Cr.ryanae              ....................KYVFLGDYIDF...GELSLEIICLLFSLKILYPDRIFLL.
Cr.bovis               ....................KYVFLGDYIDF...GGFSLEIICLLFSFKILYPDRIFLL.
Cyc.cayetanensis       ....................SLLFLGDYVDR...GHFSCEVLLLLLSFKVLYPNRVWLL.
Cys.suis               ....................DFLFLGDYVDR...GECSLEVILILFSLKILFPSRVFLL.
Cyt.felis              .......NNLNLKC...IPIKYIFISGFINNN..NKINLEYLLFIFSLKILFPYHIYFI.
E.tenella              ....................SLLFLGDFVDR...GIFSCEVLLLLLSFKVLFPDKVWLL.
E.falciformis          ....................SLVFLGDYVDR...GQFSCEVLLLLFALKVLYPSHVWLL.
H.hammondi             ....................DFLFLGDYVDR...GPCSLEVLLLLFSLKVLSPHRVFLL.
N.caninum              ....................DFLFLGDYVDR...GPCSLEVLLLLFSLKVLSPNRVFLL.
P.falciparum           N...............DNDIKYVFLGNLINR...GNYSLEVICLLFSLKILFPKHIYLL.
P.praefalciparum       N...............DNDIKYVFLGNLINR...GNYSLEVICLLFSLKILFPKHIYLL.
P.reichenowi           N...............DNDIKYVFLGNLINR...GNYSLEVICLLFSLKILFPKHIYLL.
P.blacklocki           N...............DNDIKYIFLGNLINR...GNFSLEVICLLFSLKILFPKHIYLL.
P.billcollinsi         N...............DNDIKYIFLGNLINR...GNYSLEVICLLFSLKILFPKHIYIL.
P.adleri               N...............DNDIKYIFLGNLINR...GNYSLEVICLLFSLKILFPKHIYLL.
P.gaboni               N...............DNDIKYIFLGNLINR...GNYSLEVICLLFSLKILFPKHIYLL.
P.gallinaceum          N...............ENDVKYVFLGNYVNR...GDYSLEVICFLFSLKILFPKHIYLL.
P.vivax                TSVSVGREKEAAPHTGSNDVKYVFLGNYLNR...GKHSLEVICLLLSLKVLFPKHIYLL.
P.fragile              H...............EKDVKYVFLGNYVNR...GNHSLEVICLLLSLKILFPKHIYLL.
P.knowlesi             H...............DKDIKYIFLGNYINR...GDYSLEVICLLLSLKILFPKHIYLL.
P.coatneyi             Q...............DNDVKYVFLGNYINR...GKHSLEVICLLLSLKVLFPKHIYLL.
Th.parva               ...KTGTLNFKNNNCFQFNCSYLFFGGFVSDS..SSFCLEFVLLLFALKVLFPFHVYFL.
Th.annulata            ...KNGTLNFKNNTWFQFKSSYIFFGGFVSEL..SSFCLEFVLLLFSLKVLFPFHVYFL.
Th.orientalis          .......ADFKN..WDDFDSFYVFMGGFASES..SPYALEFALLLFAMKVLFPYHVHFL.
Th.equi                .......CNNDQSSPMGEPVISIFVGNFTSDT..RVFSLEFLTFLFSLKILFPRHVYFL.
To.gondii              ....................DFLFLGDYVDR...GPCSLEVLLLLFSLKVLSPHRVFLL.
V.brassicaformis       ....................NYLFLGDYVDR...GAFSLEVVLLLFALKIQYPESVYLL.
S.cerevisiae.PPT1      .....................YLFNGDFVDR...GSWSCEVALLFYCLKILHPNNFFLN.

                           120       130       140       150       160       170
B.sp.xinjiang          .RGGREARHRDYKSPLFREICTKLRDNARMLKLGN.DEALLVQSAHELYHRIYDVFENLS
B.bigemina             .RGRREARRRDYKSALFREIYAKLSGNARQLKLAD.DEALLLQSAQELYHRIYDVFEHLS
B.ovata                .RGRREARRRDYKSALFREIYTKLSDNARQLKLAE.DEALLLQSAQELYHRIYDVFEHMS
B.divergens            .RGRREARRRDYKSALFQEIHSKLARKAAFLKLSN.DDALLVQSAHELYHRIQDVFENMS
B.bovis                .RGSRESTHRNYSSALFRELYSKLRTNAVALKLGD.DDALLVQSAHELYHRICDVFENLP
B.microti              YYEKSSNPIDTYNCHLYNDILNKLSKYHNNFNLQS.DKALISQCAKELLNKIIDIFDVMP
Be.besnoiti            .RGNHEDRQMNRDYGFFEELQRKLG.................EASEMLWEKSNDVFDLLP
Ch.velia               .RGNHEDRAMNANYGFKAECQSKLGANE................GESVWQRVNDVFEFLP
Cr.muris               .RGNHEDPLMNLSYGFHTECVRKYGSHY................GHLLWERVNDVFEFLS
Cr.andersoni           .RGNHEDPLMNLSYGFHTECVRKYGSHY................GHLLWERVNDVFEFLS
Cr.ubiquitum           .RGNHEDSSINSTFGFHLECKQKFGVN.................GEAVWERINDAFEFLS
Cr.meleagridis         .RGNHEDASVNLTNGFHFECKQKFGVD.................GEAVWERINDAFEFFS
Cr.hominis             .RGNHEDASVNLTYGFHFECKQKFGMN.................GETIWERINDAFEFFS
Cr.parvum              .RGNHEDASVNLTYGFHFECKQKFGIN.................GEAIWERINDAFEFFS
Cr.tyzzeri             .RGNHEDASVNLTYGFHFECKQKFGMN.................GEAIWERINDAFEFFS
Cr.sp.chipmunk         .RGNHEDASINSTSGFHLECKQKFGMN.................GEAIWERINDAFEFFS
Cr.ryanae              .RGNHEDVSINISSGFLDECNKKFGSN.................GQFLWERINDVFEFLS
Cr.bovis               .RGNHEDISINVSSGLLDECNKKFGSN.................GQFLWERINDVFEFLS
Cyc.cayetanensis       .RGNHEDRPMNYIYGFFSECVCKFGG................TEGEILWKRSNSLFDFLP
Cys.suis               .RGNHEDRAMNADYGFAAEIEKKLGVYGGDF...........MKARLVWQCANDVFDFLP
Cyt.felis              .CGDYEYNLIINKLNLYNNIYNILINNYNKLKLIN.NEIILIQSTNELINKIINIFNYMS
E.tenella              .RGNHEDRPMNYIYGFYAECVSKLGG................TEGEALWKRANSLFEFLP
E.falciformis          .RGNHEDRPMNFIYGFYSECLLKFGG................IEGETLWKRSNSLFDFLP
H.hammondi             .RGNHEDRQMNRTYGFLQELERKLGG................AASACVWEKANAVFDLLP
N.caninum              .RGNHEDRQMNRVYGFLEELERKLGG................DASTRVWEKANEVFDLLP
P.falciparum           .RGNHEDRLFNYIYGFYKDIEIKMKTNMEYMGIINYQEQVISAHSYELFNRINDVFEFFP
P.praefalciparum       .RGNHEDRLFNYIYGFYKDIEIKMKTNMEYMGIINYQEQVISAHSYELFNRINDVFEFFP
P.reichenowi           .RGNHEDRLFNYIYGFYKDIEIKMKTNMEYMGIINYQEQVISAHSYELFNRINDVFEFFP
P.blacklocki           .RGNHEDRLFNYIYGFYKDIEIKMKTNMEYMGIINYQEQVISAHSYELFNRINDVFEFFP
P.billcollinsi         .RGNHEDRLFNYIYGFYKDIEIKMKTNMEYMGIINYQEQVISAHSYELFNRINDVFEFFP
P.adleri               .RGNHEDRLFNYIYGFYKDIEIKMKTNMEYMGIINYQEQVISAHSYELFNRINDVFEFFP
P.gaboni               .RGNHEDRLFNYIYGFYKDIEIKMKTNMEYMGIINYQEQVISAHSYELFNRINDVFEFFP
P.gallinaceum          .RGNHEDRLFNYIYGFYKDIEVKMKMNIETMGLINYQNEVIVAHSYELFNRINDALEFLP
P.vivax                .RGNHEERLLNYVNGFYADIEKKMKRNIKTAGLIKYQEEVIEAHAYELFNRVNDVLEFLP
P.fragile              .RGNHEERLFNYVHGFYADIENKMERNGNRVGLIRYQGEVIQAHAYELFNRINDVLEFLP
P.knowlesi             .RGNHEERLFNYVHGFYGDIEKKMERNIKRGGLIRYQGEVIQAHAYELFNRINDALEFLP
P.coatneyi             .RGNHEERLFNYVHGFYADIEKKMERNIRKAGLIRYQGEVIQAHAYELFNRINDALEFLP
Th.parva               .RSSKDERDYTNNAGFYDEIYRKLSTNYDSLKLLN.DETLLLQCSKELFHKINDVFEYMP
Th.annulata            .RSPKDERDYTNNVGFYDEIYRKLSTNYESLKLPN.EETLLLQCSKELFHRINDVFEYMS
Th.orientalis          .RSSKDERNATNNAGFYHQIYMKLSENHAALRLEN.DETLLLQCAKELFHRVNDVFEFMS
Th.equi                .RSGKDNRLYNNKNGFYNEIYNTLSGNVHSLNFPS.EESLILQNARELFHRINDVFEFIS
To.gondii              .RGNHEDRQMNRTYGFLEELERKLGG................AASACVWEKANAVFDLLP
V.brassicaformis       .RGNHEDRILNKSYGFGSECVRKLGAS.................GDGLWELINDVFEFLP
S.cerevisiae.PPT1      .RGNHESDNMNKIYGFEDECKYKYSQR....................IFNMFAQSFESLP
```

```
                         180       190       200       210
B.sp.xinjiang        LAACISER.ILCIHGSLSKKFCSLDHLSNIPKPIVIS.SSVKDDNQ..............
B.bigemina           LAAIVDER.VLCVHGALSKAFRSVEQLARVRKPVIVEQTSVKGDNR..............
B.ovata              LAALLDER.VLCVHGALSKAFRSVEQLSQLRKPIIVERTSVKGDNR..............
B.divergens          ISACIEDR.VLCLHGSLSKAFRSIEQL.EISKPISTSKTNCRTRTG..............
B.bovis              LSACISDR.VLCLHGSLSPSFSSLNSLATIPKPLVLS.SSRDD.................
B.microti            KGALVDQS..ILVKGVPSDCDGVFGKYSMNKY............................
Be.besnoiti          LAAFVPTAGLFCLHGCLGDSIESINDLRSIERPLHVSALNLG.....QSLSARDLENVAA
Ch.velia             LAALVEDS.VLCVHGGIGSSVQSLADLQGIPKPISV.ISEIKEDT......TMQQRI...
Cr.muris             LGIVVEDQ.ILCVHGGIGKNIQTLDDIKDIPKPIHVSSDCFYDNSK..HEFETVNACNAY
Cr.andersoni         LGIVVEDQ.ILCVHGGIGKNIQTLDDIKDIPKPIHVSSDCFYDNSK..HEFETINACNSY
Cr.ubiquitum         ISALIDHQ.VLCLHSGIGKSIKNIEHISDVPKPIHVKSEELLNKN......NYCNRLSVS
Cr.meleagridis       ISALIDDQ.VLCIHSGIGKSIKSIENLSGIPKPIHVKSEELINKN......DFNNKLDES
Cr.hominis           ISALIDDQ.VLCLHSGIGKSIKSVANLSGIPKPIHVKSEELINKS......DCNNRLAES
Cr.parvum            ISALIDDQ.VLCLHSGIGKSIKSVENLSGIPKPIHVKS.ELINKS......DCNNRLSES
Cr.tyzzeri           ISALIDDQ.VLCLHSGIGKSIKSVENLSGIPKPIHLKSEELINKS......DCNNRLSES
Cr.sp.chipmunk       ISALIDDQ.VLCLHSGIGKSIKKIEHLSGIPKPIHVKSEELFNRI......DFNNRLVGS
Cr.ryanae            LAILINDS.IMCLHSGIGKNIKKLEHLENISKPIHVSS.DFLIRE......NFQNQLENK
Cr.bovis             LAVLVNDR.ILCLHSGIGKSIKKLEHLENISKPIHVSS.DLLIRG......TLQSSLENA
Cyc.cayetanensis     LAATVRSAGLILLHGGIGDSIQAPQQLRDIRKPITVP.QHVD.....RSLKETNPAVLKA
Cys.suis             LGAHLPAARIFCLHGCLGNSIRLIDDLRGLTRPIQVAGTDIDRKALQGIPSSSPFSSEET
Cyt.felis            IAIYINNN.ILCINGLIS...PQFNNLNQLYIKKPIGNNL....................
E.tenella            LAAVARADGLFFIHGGIGDSITSLQQLKELQKPITVP.QHVD.....RSQKDNDDTVMRA
E.falciformis        LAAAVPSAGVLLLHGGIGDSIETVQQLKGIPKPITVP.QHVD.....RSLKQSSAAVLRA
H.hammondi           LAALVPAAAVVCLHGCLGDSIEKVEDLRAIRRPLDVCAIQVE.....GPES...NPLAPE
N.caninum            LAALVPVAGVFCLHGCLGDSIETVDDLRVIQRPLDISVLQLD.....GVDAGARGASAAD
P.falciparum         LCVLLDKN.ILCIHGGIGDSVMTISDYQHIRKPILIPQNVDR.........YSNNKSEHV
P.praefalciparum     LCVLLDKN.ILCIHGGIGDSVMTISDYQHIRKPILIPQNVDR.........YSNNKSEHV
P.reichenowi         LCVLLDKN.ILCIHGGIGDSVMTISDYQHIHKPILIPQNVDR.........YSNNKSEHV
P.blacklocki         LCLLLDKN.ILCIHGGIGDSVMTISDYQHIHKPILIPQNVDR.........YSNNKSEHV
P.billcollinsi       LCVLLDKN.ILCIHGGIGDSVMTLSDYQHINKPILIPQNVDR.........YSNNKREYI
P.adleri             LGVLLDKN.ILCIHGGIGDSVMTISDYQHIHKPILIPQNVDR.........YSNNKSERV
P.gaboni             LGVLLDKN.ILCIHGGIGDSVMSISDYQHIHKPILIPQNVDR.........YSNNKSERV
P.gallinaceum        LSALLDKN.ILCIHGGIGDSVHHISDYMNIHKPILIPQYVDR.........NNNNISENT
P.vivax              LSVLVGGN.ILCVHAGIGDSLQNVGDFAAIPKPIVIPQFVNR.........SSNDAYEHV
P.fragile            LSVLVGGN.ILCVHAGIGDSLHKVEDFGDVHKPIVVPQCVDR.........TRNGNYERV
P.knowlesi           LSVLVGQS.ILCVHAGIGDSLENIEDFSDVHKPIVVPQCVDR.........TSKNEYERV
P.coatneyi           LSVLVGGN.ILCVHAGIGDSLDNVEDFADVHKPIVVPQFVDR.........TSNGAYERV
Th.parva             VCAVLNQE.VLCVDKLTSN..FNLSKLENLNRPLTLS...NCD.KE..............
Th.annulata          LCAVLNQE.ILCVDKLTNK..FSLSKLEELNKPLNQR...NFN.KE..............
Th.orientalis        VGAVLNKE.ILCVDKLTKD..FSYKRLGKIPKPLNLA...TCTSEQ..............
Th.equi              IAAIISDK.VLCLYGTIS...SELNRVDELFVPKPI........................
To.gondii            LAALVPAAAVVCLHGCLGDSIEKVEDLRAIRRPLDVCAIQME.....GPET...DPLAPE
V.brassicaformis     LAALVDGQ.IFAIHGGIGDSIGSVDDLRDLRKPINIPIPSNLHPA......SLEQRR...
S.cerevisiae.PPT1    LATLINNDYLVMHGGLPSDPSATLSDFKNIDRFAQPPRDGAF..................

                          220       230                 240       250
B.sp.xinjiang        .....AMVAYTNLHTRNALF................SSLSPVAYDGSEDQFHVKFTEDAM
B.bigemina           .....SRGAYANVHVRNACF................GTLSDTVADASGEQFHADFTEREL
B.ovata              .....SLGAYSNVHVRNAFF................GTLSDAVPNGSDEQFHVDFTEKEL
B.divergens          .....VTGFEGNTHLQHALF................GTFSPASEDVEDDWEYPNFTQEEF
B.bovis              ......IGYCSNIHARHALL................GTLS...SDDDANRFHVEFNKADM
B.microti            ......................................................IPRLTI
Be.besnoiti          NR...VMDCLWSDPE.............RPAERGEDAVG..PASPRG..GHVVRSSAAFI
Ch.velia             .....VLDCLWSDPTEND..............SVTGTHPSPRGQ......NTCRFGPDRV
Cr.muris             NVERKILDCLWSDPIDDD.....INDEKY.LYNETDVDCITNNSDNENRVNVYNYNCKDI
Cr.andersoni         NVERKILDCLWSDPIDDD.....INDEKY.LYNETDVDCITNNSDNENRVNVCNYNCKDI
Cr.ubiquitum         DR..RVFDCLWSGIIEKS....NFDDSHN.HLNEADVRNTRDADIFE..QNTCCFDSYDI
Cr.meleagridis       DR..YVLECLWSEMIDQT....SFYDSQN.FINETDVEHTRDAEIFE..KNMYFYDSYDV
Cr.hominis           DR..YVFECLWSEMIDQT....SFDDSHN.FINETDVEHTRDAEIFE..KNMYCYDSYDV
Cr.parvum            DR..YVFECLWSEMIDQT....SSDDSHN.FINETDVEHTRDAEIFE..KNMYCYDSYDV
Cr.tyzzeri           DR..YVFECLWSEMIDQT....SFDDSHN.FINETDVEHTRDAEIFE..KNMYCYDSYDV
Cr.sp.chipmunk       DR..CVFDCLWSEIIDQA....SFYSSRNDFINETDAEHTRDVDIFE..KNIYCYDSYDI
Cr.ryanae            EETVRILDCLWSEIIDSNNSVYNFNENKE.CINETDVEYN.DENSES..IKYFKFDTKDV
Cr.bovis             DETMRILDCLWSEIIESNDSICSPSESKE.CINETDVEYN.DESSEN..LKRFKFDMKDV
Cyc.cayetanensis     CQ...VLDCLWSDPL.............TADEDGAAPTS..DASPRG..KNTVRFRESAL
Cys.suis             EKMCLVLDCLWSDPE.............RPPDNKEDVAG..PDSPRG..AHAIRSSAEFI
Cyt.felis            .......NNIFINDALYGHF................INYDSINFPKCNNG.LNINNLNTS
E.tenella            CR...IIDCLWSDPV...............SPEEGEGEKTESVKSSRG..CNTV......
E.falciformis        CQ...VVDCLWSDPINEV.........IEASQQGDNPEEGQDESPRG..KNTVRFGPSRL
H.hammondi           RK...VLACLWSDPE.............RPPERAEDVDG..PTSPRG..AHVVRSSQAFI
N.caninum            HK...VLDCLWSDPE.............RPPERAEDAAG..PASPRG..GHVVRSSAAFI
P.falciparum         KK..IIIDTLWSDPINYN.....DELDLQLLK..NSSTYDIIPSRRG..NITFKFGKHRL
P.praefalciparum     KK..IIIDTLWSDPINYN.....DELDLQLLK..NSSTYDIIPSRRG..NITFKFGKHRL
P.reichenowi         KK..IIIDTLWSDPINYN.....DELDLQLLK..NSSTYDIIPSRRG..NITFKFGKHRL
P.blacklocki         KK..IIIDTLWSDPINYN.....DELDLQLLK..NSSTYDIIPSRRG..NITFKFGKHRL
P.billcollinsi       KK..IIIDTLWSDPINYN.....DELDLQLLK..NSSTYDIIPSKRG..NITFKFGKHRL
P.adleri             KK..IIIDTLWSDPINYN.....DELDLQLLK..NSATYDIIPSRRG..NITFKFGKHRL
P.gaboni             KK..IIIDTLWSDPINYN.....DELDLQLLK..KSATYDIIPSRRG..NITFKFGKHRL
P.gallinaceum        KK..IIIDTLWSDPINYN.....DEQDLFLIK..NSLHYDIIPSNRG..KITFKFGKYRL
P.vivax              QK..VIIDALWSDPINYE.....DEQDVFLLERLAHGE.DVIPSSRG..KITLKFGQRRL
P.fragile            HK..IIVDTLWSDPINYE.....DEQDMFLLQKLKSGEEDIIPSSRG..KITLKFGQKRL
P.knowlesi           QK..IIIDTLWSDPINYE.....DEEDMLLLEKVKPGG.DTIPSSRG..EITLKFGQRRL
P.coatneyi           QK..IIIDTLWSDPINYD.....DEQDMLLLEKVKPGE.DIIPSSRG..NITLKFGQQRL
Th.parva             .....IYELLFENKNPGDK.................TTNNNEGLLQDTGGYNTVKEEDND
Th.annulata          .....VYELLFDN.NSSEK.................TKNK.DCLVQDTGGYDSMDE..ND
Th.orientalis        .....VYDLLFNEDYFSFPP................CLNKHEDHQTQAPPFKATEE..VL
Th.equi              ........VIFSREVSGKIY................YYHNNVHVNEALFGSIQDDELVTS
To.gondii            RK...VLACLWSDPE.............RPPERAEDVDG..PSSPRG..SHVVRSSQAFI
V.brassicaformis     .....ILDALWSDPTDSE..............EHFGTIASQRGN......NTCRFGPDRV
S.cerevisiae.PPT1    ......MELLWADPQEAN.....................GMGPSQRG...LGHAFGPDIT
```

```
                     260       270       280       290          300       310
B.sp.xinjiang        IRCLDLAGVSLLVTSGSVADCGYSYVYGDRVLQLG....GCTAGGTYSAALLREQRSMH.
B.bigemina           AYCMRRGGVEMLITAGPPTERGYSYAYGDRVLQLG....GYTPGGIYSAALLREQR....
B.ovata              AYCMKRGGIEMLITAGPTTESGYSYAFGDRVLQLG....GYTPGGIYTAALLKEQRSTQ.
B.divergens          TSSLRAAGISMLITAGSTTERGYNYLYGERVLQLG....GCTPGGIYSAALLKEHRNMH.
B.bovis              IASLDLSGISLLVTTGCTAECGYCYMHDERVLQLG....GCTAGGVYSAALLKEHRNLH.
B.microti            IPHHSDQSTSLVSLSENLASLSNPSGCYNRVIPLTNN....SKDGLEAASLFISGD....
Be.besnoiti          ESFLDRNRLALLVRGHECVAPGYCYDLGGRCLTLFSASNYCETAQNDGAALHVYREE...
Ch.velia             GAFCEKNNLQLIIRAHECVASGFEYFAGGKLLTVFSATNYCNQYGNDGAMV.........
Cr.muris             DAFMNNTGIKLIVRAHECIPNGYEYFANGKVLTLFSTTNYCNNYKNDAAMCVFMKNTQN.
Cr.andersoni         DAFMNNTGIKLIVRAHECIPNGYEYFANGKVLTLFSTTNYCNNYKNDAAMCVFMKSPQN.
Cr.ubiquitum         EDFMNKNSIKLIIRTNDYCKGGYGYNANGRVVSLTSATNFCNNSCNDAAVLVITRGVD..
Cr.meleagridis       EDFMNTNSIKLIVKTNDYCKRGYGYNANGRVVSLNSATNFCNNFCNDAAALVITGCFE..
Cr.hominis           EDFMNTNSIKLIVKTNDYCKRGYGYNANGRVVSLNSATNFCNNFCNDAAVLVITRCFD..
Cr.parvum            EDFMNTNSIKLIVKTNDYCKRGYGYNANGRVVSLNSATNFCNNFCNDAAVLVITRCLD..
Cr.tyzzeri           EDFMNTNSIKLIVKTNDYCKRGYGYNANGRVVSLNSATNFCNNFCNDAAVLVITRCFD..
Cr.sp.chipmunk       EEFMNKNSIKLIVKTNNYCKRGYEYSANNRVVSLNSATNFCNHFSNDAAVLVITRGFD..
Cr.ryanae            VNFISNNNIELIVRTSENCRNGCESHANDKIITVTSAANTRGINNSCVSSLVITYNN...
Cr.bovis             SNFISSNDIELIVRTSEYCKNGCESHGNGKIITVTSTANARGIRNDCISALVIAYNNN..
Cyc.cayetanensis     RSFLAANSLQLLIRAHECVSGGAFYACGALCLTLFSATNYCDRFGNDGAAAQLYRDP...
Cys.suis             REFLKRNQLHLLIRAHECVLPGYCYDLDGGCLTLFSASNYCGRYQNDGAALHVYREEELV
Cyt.felis            FKLTG...IKILITNNRKLKSGFKYYFNHKILQLSSHLIHDDDDDKNATIVIVNKDDNN.
E.tenella            ............................................................
E.falciformis        SAFLAANNYKLLIRGHECVSRGAFFACGSRCLTLFSATNYCNRFANDGAAAQLYVHP...
H.hammondi           EAFLERNQLALLVRAHECVAPGYCYDLGGRCLTLFSASNYCGTAKNDGAALHIYREE...
N.caninum            EAFLERNRLALLVRGHECVAPGYCYDLGGRCLTLFSASNYCGTANNDGAALHIYREE...
P.falciparum         NDFLKNNQLKLIIRGHECVQEGYKYSYKRKLLTLFSAKNYCNKYNNNASNAFIVRKNK..
P.praefalciparum     NDFLKNNQLKLIIRGHECVQEGYKYSYKRKLLTLFSAKNYCNKYNNNASNAFIVRKNK..
P.reichenowi         NDFLKNNQLKLIIRGHECVQEGYKYSYKRKLLTLFSAKNYCNKYNNNASNAFIVRKNK..
P.blacklocki         NDFLKNNQLKLIIRGHECVQEGYKYSYKRKLLTLFSAKNYCNKYKNNASNAYIVRKNK..
P.billcollinsi       NDFLKNNKLKLIIRGHECVQEGYKYSYKRKLLTLFSAKNYCNKYNNNASNAFIVRKNK..
P.adleri             NDFLKNNQLKLIIRGHECVQEGYKYNYKRKLLTLFSAQNYCNKYKNNASNAFIVKKNK..
P.gaboni             NDFLKNNQLKLIIRGHECVQEGYKYNYKRKLLTLFSAQNYCNKYKNNASNAFIVKKNK..
P.gallinaceum        YNFLKNNKLKMIIRGHECVHEGYKYTFKKKLLTLFSARNYCKKYKNNASNAFIIKKGK..
P.vivax              SSFLRRNKLKMLIRGHECVQEGFRYSYGRRLLTLFSATNYCGRHGNDAANAFIVRRGS..
P.fragile            SSFLKKNKLKMVIRGHECVQEGYRYGYSRRLLTLFSATNYCGRYGNDAANAFIVKRGK..
P.knowlesi           SCFLKKNKLKMIIRGHECVQEGYRYSYNRRLLTLFSAKNYCNSHGNDAANAFIVKRGK..
P.coatneyi           SSFLKRNKLKMVIRGHECVQEGYRYGYNRRLLTLFSATNYCNRHGNDAANAFIVKRGK..
Th.parva             FGLGEAKNFKMVICSSDSLDTGFKYTFNNKVLLLSTNFNRKNVERVATYLSINKRKLTH.
Th.annulata          FDLGESKNFNIVICSSDSLDSGFKYTFNNKVLLLSTNFIRKNQERVATHLSIDKKK....
Th.orientalis        WELGDASRFRMVICSSDKLDAGFKYLLDNRVLLLSTNFLRKSEGRVASSLLATKNK....
Th.equi              LKKAG...IKMLISCDENLRSGYEYSHNSTVLRISTKFK....DDTVSTLVLVRSDK...
To.gondii            EAFLERNQLALLVRAHECVAPGYCYDLGGRCLTLFSASNYCGTANNDGAALHIYREE...
V.brassicaformis     MDFCERNQLKMVIRAHECVKYGYEWFARNRLLTVFSASNYCNAFANDAAAVVLRRDAST.
S.cerevisiae.PPT1    DRFLRNNKLRKIFRSHELRMGGVQFEQKGKLMTVFSAPNYCDSQGNLGGVIHVVPGHGIL

                               320
B.sp.xinjiang        ......YFITHRSLP......
B.bigemina           .....................
B.ovata              ......YVITHRSIK......
B.divergens          ......YFLIHRSIQ......
B.bovis              ......YFITHRSLP......
B.microti            .....................
Be.besnoiti          ......EEEPGALGKPR....
Ch.velia             .....................
Cr.muris             .....DNLIHFNQIIKYK...
Cr.andersoni         .....NDLIHFNQILKYKS..
Cr.ubiquitum         .....NELIQYNQII......
Cr.meleagridis       .....SQLIQYNQII......
Cr.hominis           .....NQLIQYNQII......
Cr.parvum            .....NQLIQYNQII......
Cr.tyzzeri           .....NQLIQYNQII......
Cr.sp.chipmunk       .....NQLIQYNQII......
Cr.ryanae            .....GNLLKFIQTLKFE...
Cr.bovis             .....GDLMKFIQTLKFEE..
Cyc.cayetanensis     ......TTGSLVVHT......
Cys.suis             EGGGEEDESPHGAMIPRQP..
Cyt.felis            ......NVIIHHKTLKLKH..
E.tenella            .....................
E.falciformis        ......E..............
H.hammondi           ......DCYP.ERGRRRQ...
N.caninum            ......EPLP.NPDRPRQ...
P.falciparum         ......NIIIFNQILKYQH..
P.praefalciparum     ......NIIIFNQILKYQH..
P.reichenowi         ......NIIIFNQILKCQH..
P.blacklocki         ......NIIIFNQILKYQQ..
P.billcollinsi       ......NIVIFNQILKYQHNS
P.adleri             ......NIIIFNQILKYQQ..
P.gaboni             ......NIIIFNQILKYQQ..
P.gallinaceum        ......NIIIFNQILKSDK..
P.vivax              ......SIVIFNQIV......
P.fragile            ......SIVIFNQIL......
P.knowlesi           ......NIAIFNQIL......
P.coatneyi           ......SIVIFNQIL......
Th.parva             ......MS.............
Th.annulata          .....................
Th.orientalis        .....................
Th.equi              .....................
To.gondii            ......DCYP.ERGRRRQ...
V.brassicaformis     .....GRISTLFQVLWGG...
S.cerevisiae.PPT1    QAGRNDDQNLIIETFEAVE..
```

Supplementary Table 1: Homologs of PfPPP8 in all Apicomplexan species and two non-Apicomplexan organisms identified in this study

SUPPLEMENTARY TABLE 1

Genus	Species	Gene Name	Database and Link
Babesia	*sp.Xinjiang*	BXIN_1211	PiroPlasmaDB
Babesia	*bigemina*	BBBOND_0308690	PiroPlasmaDB
Babesia	*ovata*	BOVATA_005300	PiroPlasmaDB
Babesia	*divergens*	Bdiv_035650	PiroPlasmaDB
Babesia	*bovis*	BBOV_III005730	PiroPlasmaDB
Babesia	*microti*	BmR1_04g07705	PiroPlasmaDB
Besnoitia	*besnoiti*	BESB_052670	ToxoDB
Chromera	*velia*	Cvel_26242	CryptoDB
Cryptosporidium	*muris*	CMU_013180	CryptoDB
Cryptosporidium	*andersoni*	cand_011090	CryptoDB
Cryptosporidium	*ubiquitum*	cubi_00544	CryptoDB
Cryptosporidium	*meleagridis*	CmeUKMEL1_16435	CryptoDB
Cryptosporidium	*hominis*	Chro.20178	CryptoDB
Cryptosporidium	*parvum*	cgd2_1640	CryptoDB
Cryptosporidium	*tyzzeri*	CTYZ_00002404	CryptoDB
Cryptosporidium	*sp. chipmunk*	ELE39_001909	CryptoDB
Cryptosporidium	*ryanae*	FG386_002506	CryptoDB
Cryptosporidium	*bovis*	FG379_002252	CryptoDB
Cyclospora	*cayetanensis*	cyc_07137	ToxoDB
Cystoisospora	*suis*	CSUI_002264	ToxoDB
Cytauxzoon	*felis*	CF001583	PiroPlasmaDB
Eimeria	*tenella*	ETH_00036350	ToxoDB
Eimeria	*falciformis*	EfaB_PLUS_15576.g13(	ToxoDB
Hammondia	*hammondi*	HHA_269460	ToxoDB
Neospora	*caninum*	NCLIV_036950	ToxoDB
Plasmodium	*falciparum*	Pf3D7_1018200	PlasmoDB
Plasmodium	*praefalciparum*	PPRFG01_1019300	PlasmoDB
Plasmodium	*reichenowi*	PRCDC_1017600	PlasmoDB
Plasmodium	*blacklocki*	PBLACG01_1016600	PlasmoDB
Plasmodium	*billcollinsi*	PBILCG01_1017200	PlasmoDB
Plasmodium	*adleri*	PADL01_1016600	PlasmoDB
Plasmodium	*gaboni*	PGABG01_1016100	PlasmoDB
Plasmodium	*gallinaceum*	PGAL8A_00477300	PlasmoDB
Plasmodium	*vivax*	PVP01_0603400	PlasmoDB
Plasmodium	*fragile*	AK88_04161	PlasmoDB
Plasmodium	*knowlesi*	PKNH_0602400	PlasmoDB
Plasmodium	*coatneyi*	PCOAH_00015200	PlasmoDB
Theileria	*parva*	TpMuguga_02g00379	PiroPlasmaDB
Theileria	*annulata*	TA12865	PiroPlasmaDB
Theileria	*equi*	BEWA_021210	PiroPlasmaDB
Theileria	*orientalis*	TOT_020000374	PiroPlasmaDB
Toxoplasma	*gondii*	yjq463	ToxoDB
Vitrella	*brassicaformis*	Vbra_12313	CryptoDB

Supplementary Table 2: Details on the 48 co-immunoprecipitation proteins identified in both PfPPP8-smV5 and PfCINCH-smMyc consensus lists.

SUPPLEMENTARY TABLE 2

Gene Symbol	MWT(kDa)	Annotation	Unique Control (Run 1)	Total Control (Run 1)	Unique PfPPP8 (Run 1)	Total PfPPP8 (Run 1)	Unique PfCINCH (Run 1)	Total PfCINCH (Run 1)	Intensity Control	Intensity PfPPP8	Intensity PfCINCH	Lowest value
PF3D7_0407800	229.98	protein CINCH	0	0	91	351	89	294	0	4.10E+07	6.10E+07	4.10E+07
PF3D7_0704100	425	basal complex transmembrane protein 2	0	0	89	203	67	143	0	1.30E+07	1.30E+07	1.30E+07
PF3D7_1018200	253.95	serine/threonine protein phosphatase 8, putative (PfPPP8)	0	0	92	308	42	95	0	1.10E+08	1.30E+07	1.30E+07
PF3D7_1351700	151.42	inner membrane complex protein 1f, putative	0	0	78	317	68	227	0	4.80E+07	3.20E+07	3.20E+07
PF3D7_1436200	270.31	basal complex protein 1	0	0	75	191	50	97	0	2.10E+07	1.00E+07	1.00E+07
PF3D7_0704300	217.36	protein BLEB	0	0	50	128	37	78	0	9.00E+06	8.10E+06	8.10E+06
PF3D7_1014900	267.82	conserved Plasmodium protein, unknown function	0	0	44	87	16	20	0	3.10E+06	6.60E+05	6.60E+05
PF3D7_1229800	270.61	myosin J, putative	0	0	36	67	31	53	0	2.60E+06	2.50E+06	2.50E+06
PF3D7_1435600	210.58	conserved Plasmodium protein, unknown function	0	0	28	50	17	28	0	1.50E+06	1.30E+06	1.30E+06
PF3D7_0304100	61.73	inner membrane complex protein 1e, putative	0	0	23	89	19	50	0	1.30E+08	3.70E+06	3.70E+06
PF3D7_0611600	76.62	basal complex transmembrane protein 1	0	0	20	60	21	41	0	4.40E+06	3.70E+06	3.70E+06
PF3D7_1341500	58.81	Inner membrane complex suture component,putative	0	0	21	47	13	24	0	2.60E+06	2.20E+06	2.20E+06
PF3D7_1031200	41.42	MORN repeat-containing protein 1	0	0	14	43	14	28	0	7.60E+06	4.20E+06	4.20E+06
PF3D7_0506900	86.6	Rhomboid protease ROM4	0	0	17	30	10	13	0	6.70E+05	1.30E+06	1.30E+06
PF3D7_0525800	34.32	inner membrane complex protein 1g, putative	0	0	17	136	16	96	0	7.50E+07	5.50E+07	5.50E+07
PF3D7_1345600	43.19	inner membrane complex protein	0	0	16	74	12	35	0	7.40E+06	6.90E+06	6.90E+06
PF3D7_0214700	35.68	conserved Plasmodium protein, unknown function (PF3D7_02147(	0	0	15	24	13	17	0	1.30E+06	1.20E+06	1.20E+06
PF3D7_1126700	110.05	Autophagy-related protein 23, putative	0	0	14	23	9	13	0	9.00E+05	6.30E+05	6.30E+05
PF3D7_1342600	92.22	Myosin-A	0	0	12	25	13	21	0	7.00E+05	1.40E+06	7.00E+05
PF3D7_0515700	51.77	Glideosome-associated protein 40	0	0	14	29	8	14	0	3.30E+06	1.70E+06	1.70E+06
PF3D7_0618000	92.7	conserved Plasmodium protein, unknown function	0	0	11	18	11	17	0	1.20E+06	1.80E+06	1.20E+06
PF3D7_1019100	229.03	Unknown, also pulled down in CINCH IP	0	0	11	13	4	4	0	1.60E+05	6.80E+04	6.80E+04
PF3D7_1003600	32.63	inner membrane complex protein 1c, putative	0	0	12	74	10	33	0	2.00E+07	1.50E+07	1.50E+07
PF3D7_0320800	49.38	ATP-dependent RNA helicase DDX6	0	0	7	9	5	8	0	4.10E+05	5.10E+05	4.10E+05
PF3D7_0806800	122.92	Vacuolar proton translocating ATPase subunit A,putative	0	0	6	11	6	9	0	2.60E+05	3.30E+05	2.60E+05
PF3D7_1229300	117.41	conserved Plasmodium protein, unknown function	0	0	10	12	4	5	0	1.70E+05	1.50E+05	1.50E+05
PF3D7_1315300	56.31	conserved Plasmodium protein, unknown function	0	0	10	23	5	8	0	1.30E+06	4.10E+05	4.10E+05
PF3D7_1227700	91.24	conserved Plasmodium protein, unknown function	0	0	9	13	2	2	0	2.70E+05	1.30E+06	2.70E+05
PF3D7_0822900	138.16	conserved Plasmodium protein, unknown function	0	0	6	9	5	9	0	2.80E+05	4.50E+05	2.80E+05
PF3D7_1304200.1	54.86	CorA-like Mg2+ transporter protein, putative	0	0	8	10	4	5	0	3.10E+05	1.60E+05	1.60E+05
PF3D7_1142100	324.63	conserved Plasmodium protein, unknown function	0	0	6	6	4	6	0	7.50E+04	7.90E+04	7.50E+04
PF3D7_0423500	42.59	glideosome associated protein with multiple membrane spans 2	0	0	7	64	6	38	0	2.10E+07	1.60E+07	1.60E+07
PF3D7_1409400	30.52	conserved Plasmodium protein, unknown function	0	0	8	64	7	25	0	2.00E+07	1.10E+07	1.10E+07
PF3D7_0914400	28.4	conserved Plasmodium protein, unknown function	0	0	7	37	7	23	0	3.40E+06	2.30E+06	2.30E+06
PF3D7_1222700	23.62	Glideosome-associated protein 45	0	0	8	20	4	12	0	1.90E+06	1.30E+06	1.30E+06
PF3D7_0217500	60.76	Calcium-dependent protein kinase 1	0	0	6	11	6	7	0	3.50E+05	3.10E+05	3.10E+05
PF3D7_1429800	159.87	Coatamer beta subunit, putative	0	0	2	2	2	2	0	1.40E+04	8.20E+04	1.40E+04
PF3D7_1308000	99.95	conserved Plasmodium protein, unknown function	0	0	6	11	3	5	0	8.30E+04	5.30E+04	5.30E+04
PF3D7_1446600	19.3	Centrin-2	0	0	5	10	6	7	0	6.00E+05	3.30E+05	3.30E+05
PF3D7_0109000	25.46	Photosensitized INA-labeled protein PHIL1,putative	0	0	6	45	3	7	0	2.80E+06	1.20E+06	1.20E+06
PF3D7_1012900	43.49	Autophagy-related protein 18, putative	0	0	5	7	6	10	0	2.60E+05	2.00E+06	2.60E+05
PF3D7_1423700	183.46	conserved Plasmodium protein, unknown function	0	0	5	8	4	6	0	9.60E+04	1.30E+05	9.60E+04
PF3D7_1356800	475.42	Serine/threonine protein kinase, putative	0	0	5	6	3	3	0	4.40E+04	2.70E+04	2.70E+04
PF3D7_0522600	55.53	Inner membrane complex protein	0	0	4	7	4	5	0	9.90E+05	1.70E+05	1.70E+05
PF3D7_1037500	81.47	Dynamin-like protein	0	0	3	4	3	3	0	6.30E+04	9.80E+04	6.30E+04
PF3D7_1310700	16.52	RNA-binding protein	0	0	4	45	3	26	0	1.40E+07	1.10E+07	1.10E+07
PF3D7_1246400	23.48	Myosin A tail domain interacting protein	0	0	3	7	4	10	0	3.30E+05	9.00E+05	3.30E+05
PF3D7_1323100	21.59	60S ribosomal protein L6, putative	0	0	3	5	3	5	0	1.70E+05	2.70E+05	1.70E+05

Supplementary Table 3: Primers and Synthetic DNA blocks used in this study.

SUPPLEMENTARY TABLE 3

Oligo Name	Oligo Sequence
oJDD56	ACACTTTATGCTTCCGGCTCGTATGTTGTG
oJDD512	TGCACCTTGAAGCGCATGAACTCC
oJDD2933	CTGCTGCTGAGTACTATCAAGTC
oJDD3927	GCTCTAAAACtgagatggtttgactcgattcAATATTATATACTTAATATGAAATATGTGC
oJDD3928	TATAATATTgaatcgagtcaaaccatctcaGTTTTAGAGCTAGAAATAGCAAGTTAAAATAAG
oJDD4293	GCTCTAAAACctgattttagttctggtcgtcAATATTATATACTTAATATGAAATATG
oJDD4571	TATAATATTgatgttaggaaaatcacaagtGTTTTAGAGCTAGAAATAGCAAGTTAA
oJDD4572	GCTCTAAAACacttgtgattttcctaacatcAATATTATATACTTAATATGAAATATG
oJDD4616	gtcgctcgagATGGTTTCGAAAGGAGAGGAGGAT
oJDD4617	cgaccgtacgttaCTACTTATAGAGTTCATCCAT
oJDD4682	TATTgTACCTTGAAATCATTAAGAG
oJDD4683	AAACCTCTTAATGATTTCAAGGTAc
oJDD4688	CTAATATATTATCGAGACccgcgggatatcaggcctGATGAAAAAGGAGATAATGAAAAAG
oJDD4689	GGcGGtAgGCAtTGCATtTGCATtTTATTactTGAgTCCTTgATgTTATTgTCtCCaCgcAAgctcTTgAgtGTcGTAACATTATTCCTTTGATCCTTG
oJDD4690	CCATccatggtGAgTCgTTgATcTTtTCcAAtgacTTcTGgAAtGTgATcTTcGTcTGtGGcTTcGGaTCtGGcGGtAgGCAtTGCATtTGCATtTT
oJDD4691	CTCCTTTTTCATCaggcctgatatcccgcggGTCTCGATAATATATTAGAGATGGTC
oJDD4692	CATCGCggccgcaGACACCTTAATGATATGTGTGATTG
oJDD4693	TATTgAAGGAAAATATTCAATTACA
oJDD4694	AAACTGTAATTGAATATTTTCCTTc
oJDD4695	TATTgCAAGTTGATAAAGAACATTA
oJDD4696	AAACTAATGTTCTTTATCAACTTGc
oJDD4699	CATCGCggccgcCCTTTATGTTAATTGTTTTTTGTTCACATG
oJDD4700	GTATAACATTTCTAAAGaggcctgatatcccgcggCAAAATGCTCGAGAAGCCTTAATAC
oJDD4701	CTCGAGCATTTTGccgcgggatatcaggcctCTTTAGAAATGTTATACGACGATTCTAAC
oJDD4702	GGCAgTCtTTgATaTTtTTgATgAgtTTaGAgTAcTGATCtAGaACgTCcTGcAAcTGtATgTTcTCtTTTTGAACATATATAAGCGATGAATTCAAGG
oJDD4703	CCATccatgggATtGATAAaTAaTTTGCgAAcTTATTtAgTTCaTCgTTAACgGCtTTCATgTCtGGGCAgTCtTTgATaTTtTTgATgAgt
oJDD4925	CGTAcccgggttaCAGATCCTCTTCGCTGATAAGTTTC
oJDD5007	CAACGCCATTAATGCTGC
oJDD5078	GTACAGGGTCTCGggagATTCTATAAACCTGTTAGGAATAAAGG
oJDD5079	GTACAGGGTCTCGagtaTTAGGATATTTTGATTCTTCAATAAACC
oJDD5398	CATGCCATGGGTAGGAATGAAAAGGTAAAAAAAGAAAAATG
oJDD5839	CTTTGTAAAATTTGCATAACTAGAAAAAGTAATACTATATC
oJDD6010	GATAGGATAAGTTTTTGAATATTTTATTATTGTTAC
oJDD6246	GTACAGGGTCTCGCATGGAGCAGAAGAACTCGACGAACATC
oJDD6247	GTACAGGGTCTCGCTTCATGGAGGGGTGCGTATTTAACTG
oJDD6248	GTACAGGGTCTCGGAAGCTCGAGCACCACCACCAC
oJDD6249	GTACAGGGTCTCGCATGGCGCTGCTGTGATGATG
oJDD6292	TATTGTGTTCTATTCACTCCGTGAT
oJDD6293	AAACATCACGGAGTGAATAGAACAC
oJDD6294	TATTGTTCATCACTTCGAAAGCAT
oJDD6295	AAACATGCTTTCGAAGTGATGAAC
oJDD6296	TATTGTTGCTTACATTGTATGACGA
oJDD6297	AAACTCGTCATACAATGTAAGCAAC
oJDD6358	CATGGCGGCCGCGAACATCAATATATATTTTTTAATATATGTTGATAATTATAGTGTCC
oJDD6359	GATCACAATTTTCGCCTGACTTGTTCAGGAGGCCTTTGGATCCCGCATTTAATATGTTATCCATTTTTCTTTTCTCTC
oJDD6360	GAGAGAAAAGAAAAATGGATAACATATTAAATGCGGGATCCAAAGGCCTCCTGAACAAGTCAGGCGAAAATTG
oJDD6361	CAGTCGCCCTCATCGTAGAGCGTCAGCAGTTGCTCATCCTTAAAGTTGTAATAATATGCTTG
oJDD6362	CAAGCATATTATTACAACTTTAAGGATGAGCAACTGCTGACGCTCTACGATG
oJDD6363	CATGCCATGGTTCGGTAATCGGCAGGATCTTCTTC
oJDD6503	CATGCCATGGATGGTGAGCAAGGGCGAGG
oJDD6504	GGAGGCCTCCCAGCCCATCGTCTTCTTCTGCATTACGGGG
oJDD6505	CCCCGTAATGCAGAAGAAGACGATGGGCTGGGAGGCCTCC
oJDD6506	CATGGGTACCTTACTTGTACAGCTCGTCCATGCCG
oJDD6545	CGATATAAAGTACGTCTTCTTGGGCGACTTCGTCGACAGGGGCAACTACTCCTTGGAG
oJDD6546	CTCCAAGGAGTAGTTGCCCCTGTCGACGAAGTCGCCCAAGAAGACGTACTTTATATCG
oJDD6547	CATGGGTCTCGCCATGGAATTTGAACAGCTGTATGACGATAAGAATG
oJDD6548	CATGGGTCTCGCGAGTAAACCAAAACCACCATTAGAATAGGCC
oJDD6549	CATGGGTCTCGCTCGAGCACCACCACCACCAC
oJDD6550	CATGGGTCTCGATGGCGCTGCTGTGATGATGATGATG
oJDD6637	CTCTTAGTTCAATATGGTAGAAAATGATAGGAAG
oJDD6638	GACCTAAAGCATCATCTGGTAAAAAATG
oJDD6762	CAGCTCTTCACCCTTCGACA
oJDD6849	GTACCGTCCTCTCTCGTGATCGAGGGAAGGATTTCAGAATTCTTAAATTGCTCAGAAATAATGGATTTGTGTGATCG
oJDD6850	ACGACGTTGTAAAACGACGGCCAGTGCCAAGCTTTCAAGAATTAATCTGATCAAACCACCCGAAAGAATTATC
oJDD6851	GTACCGTCCTCTCTCGTGATCGAGGGAAGGATTTCAGAATTCGAATTTGAACAGCTGTATGACGATAAGAATG
oJDD6852	ACGACGTTGTAAAACGACGGCCAGTGCCAAGCTTTCATAAACCAAAACCACCATTAGAATAGGCCATTG
oJDD6870	CCCCTCTATGGAAATGAGGCCATGAGAATTGTTCGAAAAAATACAATAGGTCAATTAGCTGCCCATGAATGTCTC
oJDD6871	CTCATGGCCTCATTTCCATAGAGGGGACATATTATCGATGAAATATGTTTTCTTGGGAGACTATGTTGATAGAGG
oJDD7155	CGTTGGATGGAGATATGAATCCTTCATTCC
oJDD7156	GAGAATGAAACACACGGATTTAAAGAACATAAAG
oJDD7157	GAAATTCTGGAAAGGAACCTTTGAGACAATC
oJDD7158	CATACGGATCTTTTAGATCTATGCC
oJDD7160	CAACAGATCAATGAGCTCATCC
oJDD7161	GTAGTGCATGCGCTCGCC

Gene Block Name	Gene Block Sequence
CaPfMyoJ	CTGCTGACGCTCTACGATGAGGGCGACTGCGATACCATTGACATCAGCGATAAGGCGAAGCAGTCCTATAATATTCTCTCCTGCACGCCGCTGA<br>ACCATCTCGATACGATCCTCCTGCTGATGAAGGACATCGACGAAAACAACTCGTACGCCTTTGAGGTCATGAATATTGTGAATAAGAAGAAGA<br>AGATCCTGCCGATTACCGAA
CaPfPPP8_Phosphat	GAGCAGAAGAACTCGACGAACATCAACAAGAGCCTGCCCCACTTCGGCAAGAAGGAGGACATCCAGGACAATGTCATCAACAAGGAGTTGCA<br>CATCGAGCACATCATCGAGGACAAGATCGAAAGGATCAAGAAGCTGGTGAAGATCTACCGGGAGAAGTACATCACGGACCATAGGCTGAAC<br>ACGCTGAACCAGGACATCGCCTTTAAGATCTTCACGATGTTCTACAAGGTGTGCTACGGCAAGAAAAAGCACACCTACACCTCCTTCTTCGAGA<br>AATTTCAGATCTGCACCTATAACGACATCCTCCTCTTGTGCGACGAAGTCGCCAAGTTGTTCAAGATGGAGAATAGCTTGGAGAATGTCACCTT<br>ACCCTGCAAGGTCTTCGGCGACGTCCACGGCAACTTGTTCGACCTCTTAGATTTTTTCAACTTGTACAACTGGCCGATGCACAACAACAAAAACT<br>TGTTGCTCACCGAGAGTGACTTGAGGAAGTTCGAGTACATTCACAACGACAAGGACATCCACATCGACAAGAACAAGGACATGCAGTACAAC<br>GACAACGATATAAAGTACGTCTTCTTGGGCAACTTGATCAACAGGGGCAACTACTCCTTGGAGGTCATCTGCTTATTGTTTAGCTTGAAGATCT<br>TGTTCCCGAAGCACATCTACCTCTTGAGGGGCAACCACGAGGACAGGTTGTTCAACTACATCTACGGCTTCTACAAGGACATAGAGATCAAGAT<br>GAAGACGAACATGGAGTACATGGGCATCATCAACTACCAGGAGCAGGTCATCTCCGCCCACTCGTACGAGCTGTTTAATAGGATCAACGACGT<br>CTTCGAGTTCTTTCCGTTGTGCGTCTTGTTGGACAAGAACATCCTGTGCATCCACGGCGGCATCGGCGACAGCGTCATGACCATCTCGGACTAC<br>CAGCACATCCGCAACCCCATCTTGATCCCGCAGAACGTCGACAGGTACAGCAACAACAAGAGTGAGCACGTGAAGAAGATCATTATCGACACCT<br>TATGGTCGGACCCCATCAACTACAACGACGAGCTGGACTTGCAGTTGTTGAAGAACTCGTCCACCTACGACATCATACCgTCGAGGAGGGGGA<br>ACATAACGTTTAAGTTCGGCAAGCACAGGCTCAACGACTTTTTGAAGAACAATCAGCTGAAGCTGATCATCAGGGGCCACGAGTGCGTCCAGG<br>AGGGCTACAAGTACTCGTATAAGAGGAAGTTACTGACGCTGTTCTCGGCCAAGAACTACTGCAATAAGTACAACAACAACGCGTCGAACGCGT<br>TTATCGTCAGAAAGAACAAGAACATCATCATTTTCAATCAGATCTTAAAGTATCAGCATAACTGCAACACGCTCATCAACAACGAGAACAAGCA<br>GGTGACGATCAACCAACTcCAGAAGTACAATTTCTCCAACGACAAGTCCAACGACAAGAAGACGTCCCCGTTCTTCAATAACACGCACCTCACGA<br>AAAAGACGTTCGCCGTCTCCCACGGGTACTTATCGAAGTGCGACGAGGAGTTGAACGACCAGTTAAATACGCACCCCTCCATGAAG
