# Supplementary material for: A PPP-type pseudophosphatase is required for the maintenance of basal complex integrity in Plasmodium falciparum
Source: Nat Commun. 2023 Jul 3;14:3916. doi: 10.1038/s41467-023-39435-z (PMC10317984; doi:10.1038/s41467-023-39435-z)
Supplement: Supplementary file 3 — Description of additional supplementary files [file 41467_2023_39435_MOESM3_ESM.pdf]

Description of additional supplementary files

Title: Supplementary Movie 1
Description: U-ExM of PfPPP8-smV5^{Tet}; PfCINCH-smMyc parasites stained with anti-V5 (green), anti-Myc (magenta) and NHS-ester AF405. 0.15 µm Z-stacks; PfPPP8-sufficient. Corresponds with Fig. 2d.

Title: Supplementary Movie 2
Description: U-ExM of PfPPP8-smV5^{Tet}; PfCINCH-smMyc parasites stained with anti-V5 (green), anti-Myc (magenta) and NHS-ester AF405. 0.15 µm Z-stacks; PfPPP8-deficient. Corresponds with Fig. 2d.

Title: Supplementary Movie 3
Description: 3-Dimensional rendering of time-lapse microscopy of PfPPP8-smV5^{Tet}; PfBLEB-HaloTag parasites. PfBLEB is visible in magenta; parasites were treated with AF647 HaloTag ligand under PfPPP8-sufficient conditions. Corresponds with Fig. 3c-d.

Title: Supplementary Movie 4
Description: 3-Dimensional rendering of time-lapse microscopy of PfPPP8-smV5^{Tet}; PfBLEB-HaloTag parasites. PfBLEB is visible in magenta; parasites were treated with AF647 HaloTag ligand under PfPPP8-deficient conditions. Images were taken every 20 minutes. Corresponds with Fig. 3c-d.

Title: Supplementary Movie 5
Description: 3-Dimensional rendering of time-lapse microscopy of PfPPP8-smV5^{Tet}; PfBLEB-HaloTag parasites. PfBLEB is visible in magenta; parasites were treated with AF647 HaloTag ligand under PfPPP8-sufficient conditions (additional replicate). Images were taken every 20 minutes. Corresponds with Supplementary Fig. 5d-e.

Title: Supplementary Movie 6
Description: 3-Dimensional rendering of time-lapse microscopy of PfPPP8-smV5^{Tet}; PfBLEB-HaloTag parasites. PfBLEB is visible in magenta; parasites were treated with AF647 HaloTag ligand under PfPPP8-deficient conditions (additional replicate). Images were taken every 20 minutes. Corresponds with Supplementary Fig. 5c.

Title: Supplementary Movie 7.
Description: 3-Dimensional rendering of time-lapse microscopy of PfCINCH-mNeonGreen; PfPPP8-mCherry parasites. PfCINCH is visible in green, PfPPP8 is visible in magenta. An early schizont (starts imaging ~40 hpi) is adjacent to an older schizont (start imaging ~46 hpi); as the basal complex of the latter shrinks and contracts, that of the former is built. Images were taken every 20 minutes. Corresponds with Fig. 6c-d.

Title: Supplementary Movie 8.
Description: 3-Dimensional rendering of time-lapse microscopy of an older PfCINCH-mNeonGreen; PfPPP8-mCherry schizont (additional replicate). PfCINCH is visible in green, PfPPP8 is visible in magenta. This parasite starts imaging ~46 hpi; the basal complex shrinks and contracts. Images were taken every 20 minutes. Corresponds with Supplementary Fig. 11a-b.

Title Supplementary Movie 9.
Description: 3-Dimensional rendering of time-lapse microscopy of a younger PfCINCH-mNeonGreen; PfPPP8-mCherry schizont (additional replicate). PfCINCH is visible in green, PfPPP8 is visible in magenta. This parasite starts imaging ~40 hpi; the basal complex is being constructed and expanding. Images were taken every 20 minutes. Corresponds with Supplementary Fig. 11a-b.

Title: Supplementary Movie 10 (older PfCINCH-mScarlet; PfPPP8-mNeongreen schizont)
Description: 3-Dimensional rendering of time lapse microscopy of a more mature PfCINCH-mScarlet; PfPPP8-mNeonGreen schizont. PfCINCH is visible in magenta, PfPPP8 is visible in magenta. This parasite starts imaging ~ 46 hpi; the basal complex shrinks and contracts. Images were taken every 20 minutes. Corresponds with Supplementary Fig. 12c.

Title: Supplementary Movie 11 (younger PfCINCH-mScarlet; PfPPP8-mNeongreen schizont)
Description: 3-Dimensional rendering of time lapse microscopy of a younger PfCINCH-mScarlet; PfPPP8-mNeonGreen schizont. PfCINCH is visible in magenta, PfPPP8 is visible in magenta. This parasite starts imaging ~ 40 hpi; the basal complex is being constructed and expanding, then shrinks and contracts. Images were taken every 20 minutes. Corresponds with Supplementary Fig. 12e.
