# Supplementary material for: A PPP-type pseudophosphatase is required for the maintenance of basal complex integrity in Plasmodium falciparum
Source: Nat Commun. 2023 Jul 3;14:3916. doi: 10.1038/s41467-023-39435-z (PMC10317984; doi:10.1038/s41467-023-39435-z)
Supplement: Supplementary file 15 — Source Data [file 41467_2023_39435_MOESM15_ESM.zip › source data (combined)/Source Data (Westerns).pdf]

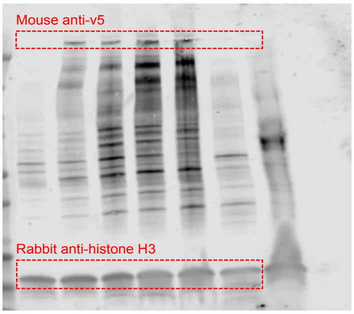

Figure 6b

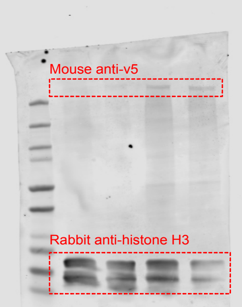

Supplementary Figure 1d

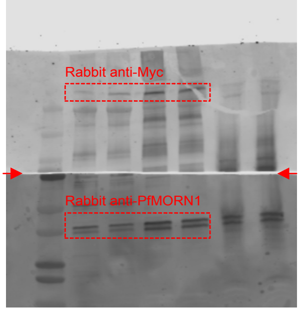

Supplementary Figure 5b (PfCINCH and PfMORN1)

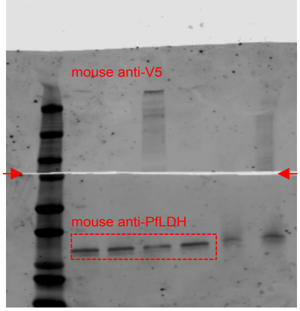

Supplementary Figure 5b (PfLDH)

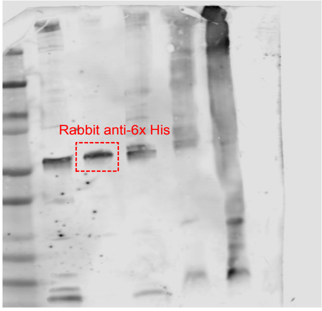

Supplementary Figure 6c, PfPPP8-6xHis

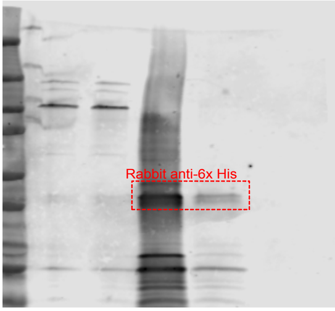

Supplementary Figure 6c, ScPPT1, CpPPP8-6xHis

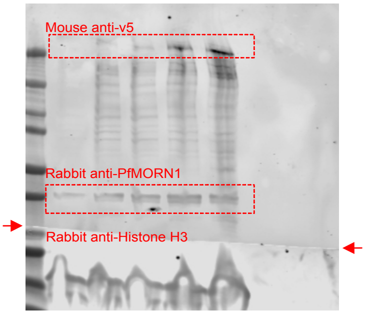

Supplementary Figure 7b, PfMyoJ and PfMORN1

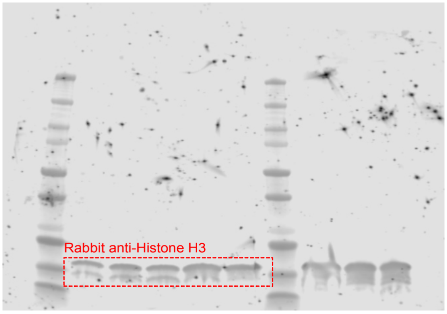

Supplementary Figure 7b, histone H3

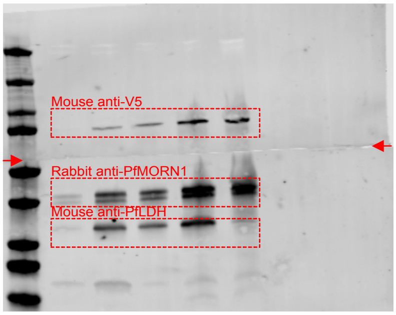

Supplementary Figure 7e

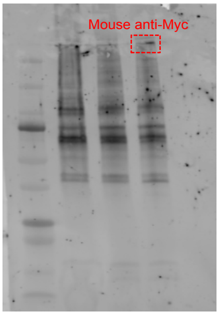

Supplementary Figure 9c, Compound 1 stall time point (PfCINCH only)

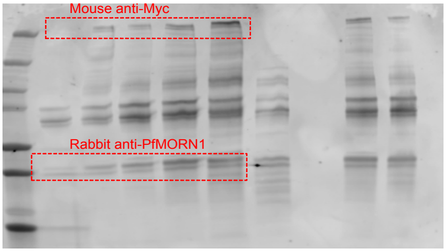

Supplementary Figure 9C, Time points 40-48 (PfCINCH and PfMORN1) and Compound 1 Stall (PfMORN1 only)

Dashed Red Boxes depict sections shown in figure(s). Red arrows depict where membrane was cut for incubation with multiple primary antibodies. Antibodies used are written on the blot in red text above each red box.
